# Molecular demultiplexer as a terminator automaton

**DOI:** 10.1038/s41467-018-03259-z

**Published:** 2018-02-23

**Authors:** Ilke S. Turan, Gurcan Gunaydin, Seylan Ayan, Engin U. Akkaya

**Affiliations:** 1 UNAM-National Nanotechnology Research Center, Bilkent University, 06800 Ankara, Turkey; 2 Department of Basic Oncology, Hacettepe University, 06100 Ankara, Turkey; 3 Department of Chemistry, Bilkent University, 06800 Ankara, Turkey

## Abstract

Molecular logic gates are expected to play an important role on the way to information processing therapeutic agents, especially considering the wide variety of physical and chemical responses that they can elicit in response to the inputs applied. Here, we show that a 1:2 demultiplexer based on a Zn^2+^-terpyridine-Bodipy conjugate with a quenched fluorescent emission, is efficient in photosensitized singlet oxygen generation as inferred from trap compound experiments and cell culture data. However, once the singlet oxygen generated by photosensitization triggers apoptotic response, the Zn^2+^ complex then interacts with the exposed phosphatidylserine lipids in the external leaflet of the membrane bilayer, autonomously switching off singlet oxygen generation, and simultaneously switching on a bright emission response. This is the confirmatory signal of the cancer cell death by the action of molecular automaton and the confinement of unintended damage by excessive singlet oxygen production.

## Abstract (web summary)

Nanorobots will likely play a major role in future therapeutic approaches. Here, the authors develop a molecular automaton based on Zinc-BODIPY complexes that can autonomously switch between singlet oxygen generation and fluorescence emission, i.e., therapeutic and diagnostic modes.

## Introduction

More than two decades after the seminal work by de Silva et al.^1^, molecule-based logic gates have reached a level of considerable sophistication^2–4^. Many examples of basic Boolean logic gates and various implementations of both combinatorial and sequential logic were reported^5–10^. While it is clear that more advanced digital designs may require novel integration mechanisms of chemical logic gates, functional equivalents of more complex information processing is still possible with simple molecules or molecular assemblies as a result of their unique characteristics. However, despite this impressive progress, the power of chemical and molecular logic gates beyond the exploratory phase is yet to be convincingly demonstrated,^11^ with what is colloquially referred to as a killer app^12^.

**Fig. 1:**
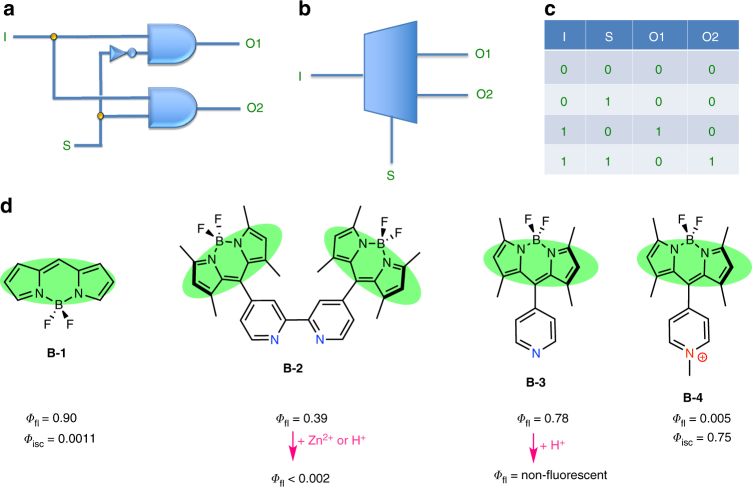
DEMUX combinatorial circuit and structures of relevant Bodipy compounds. **a** DEMUX circuit with standard logic gate symbols. **b** Isosceles trapezoid is a common symbol for the MUX/DEMUX circuits. I is the data input, S is the *select* or *address* input, and O1 and O2 are two different outputs. **c** The truth table for a 1:2 DEMUX circuit is on the right. **d** Structures of the parent Bodipy compound **B-1**^31^, and *meso*-pyridyl^22^/bipyridyl^20^ substituted derivatives, together with their respective fluorescence and intersystem crossing quantum efficiencies are demonstrated. Protonation of both **B-2** and **B-3** result in the pyridinium cation, which switches on the intramolecular charge transfer process. Coordination of Zn^2+^ ions or quaternization at the pyridine nitrogen (**B-4**) results in the same photophysical consequences

The most likely field where molecule-based information processing agents would find a niche for true utility is therapeutic medicine. We previously described various protocols to combine molecular logic gate notions with photosensitized generation of short-lived cytotoxic species, singlet oxygen^3,13,14^. Photosensitized generation of singlet oxygen in tumors, in or around cancer cells, is the essence of photodynamic therapy of cancer^15–18^. The fate of the singlet excited state (S_1_) of the photosensitizer is strongly related to the relative efficiencies and rates of photophysical processes involved. Among these, the most relevant are radiative transition from S_1_ to S_0_ (fluorescence) and intersystem crossing to T_1_ triplet state (isc). Efficiency of access to the triplet manifold is directly linked to the singlet oxygen quantum yield. Thus, considering the fact that in the photosensitization process, fluorescence and intersystem crossing (hence, singlet oxygen generation) are mutually antagonistic, and at least in principle, it should be possible to switch between these two processes. The digital equivalent is that of a demultiplexer (DEMUX) circuit^19^, which takes single data input, and uses *n* *select*/*address* inputs to switch between 2^*n*^ possible outputs (Fig. 1).

Chemical implementation of this idea relies on the photophysics of the *meso*-pyridyl (or oligopyridyl) substituted Bodipy dyes (Fig. 1d). Bodipy dyes are exceptionally versatile and chemically malleable chromophores. The reference Bodipy chromophore (**B-1**) has high fluorescence quantum yield with a small Stokes’ shift and, on excitation, would have negligible access to the triplet manifold. This is also in part the reason for higher chemical stability of this class of dyes under ambient conditions. In our earlier investigations^20^ aiming cation-responsive fluorophores (chemosensors), we observed a sharp decrease in the emission of bipyridyl-Bodipy compound (**B-2**) when an acid (trifluoroacetic acid (TFA)) or zinc perchlorate was added. Apparently, protonation of the ligand, or complexation with a +2 charged cation, transforms the ligand into a more easily reducible species, and photoinduced electron transfer from the excited Bodipy dye to the bipyridyl ligand becomes more efficient. Resulting charge transfer state (CTS) is responsible for the overall non-radiative relaxation (hence quenching). The orthogonal arrangement of the ligand and the chromophore is expected to facilitate intersystem crossing to the triplet state, as the charge recombination is typically accompanied with the population of the triplet excited state^21^. Considering recent data on the photophysics of the similar Bodipy-pyridyl ligand systems^22^, we concluded that such protonated pyridinium, or quaternized pyridinium substituted Bodipy dyes, and the Zn^2+^ complexes are likely to be more efficient generators of singlet oxygen compared to the parent compounds from which they are derived due to enhanced yield of the triplet state. Another useful aspect of this change is that it is chemically reversible; i.e, if the protonation is reversed, or charge on the Zn^2+^ ion is neutralized (even partially), a return to the original state of affairs should be expected. Thus, the addition of phosphate to the **B-2**/Zn^2+^ complex destabilizes the CT state and restores the fluorescence emission intensity.

In a demultiplexer, the *select* input would determine the choice between the possible outputs. Searching for a candidate as the *select* input, we looked into the possibility of making use of structural changes taking place in the cell membranes during apoptosis. Fluorescence imaging of apoptosis relies on the loss of membrane asymmetry (adenosine triphosphate (ATP)-dependent enzyme called flippase normally keeps phosphatidylserine inside the cell)^23,24^ as the negatively charged lipids and particularly phosphatidylserine is flipped to the extracellular side of the bilayer. The change is mediated by enzymes such as scramblase, which exposes phosphatidylserine on the cell’s surface without consuming ATP^23,25^. A selective probe for this event is the protein Annexin V, which selectively binds to phosphatidylserine. Since Annexin V can be conveniently labeled with fluorescent dyes of different emission colors, it provides a set of useful tools for detecting apoptotic cells. There is also an interest in finding simpler, non-protein reporters of apoptosis, and in most of the reported examples^26^, the part which interacts with the phosphatidylserine unit is a Zn^2+^ complex, in which the metal ion is held in place by pyridine-derived ligands, presenting coordination sites to the anionic groups of the phosphatidylserine^27^.

In this work, we present a unique molecular device based on above considerations. The molecular automaton described here initially generates singlet oxygen to trigger apoptosis in cancer cells, and then, in response to apoptotic changes in the membrane structure, shuts off singlet oxygen generation and produces emission as a result of its interaction with the exposed phosphatidylserines in the outer leaflet of the apoptotic cell membranes. This could be interpreted as the response of a molecular demultiplexer which takes light as an input and phosphatidylserines as the switch. The two alternative outputs are singlet oxygen and light.

## Results

### Operation of the automaton

**Fig. 2:**
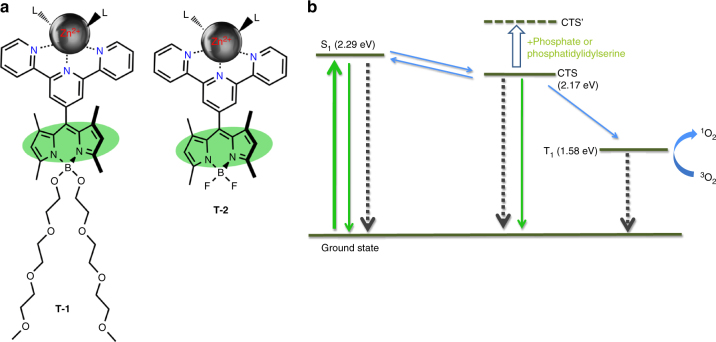
The structures of the molecular automata **T-1** and **T-2** and the Jablonski diagram depicting processes involved. **a** L indicates solvent molecules as ligands, water, or acetonitrile. Bodipy and the terpyridyl planes are orthogonal to each other due to the presence of 1,7-dimethyl substitution of the Bodipy chromophore. Double mTEG (methoxytriethyleneglycol) substitution at the boron center enhances water solubility. **b** Thicker green arrow is absorption, the other green arrows indicate radiative relaxations. Black-dashed arrows are non-radiative relaxation processes. Blue arrows indicate transitions between various excited states. CTS to T_1_ transition is particularly enhanced because of the orthogonal geometry of the terpyridyl-Bodipy diad. Energy levels were experimentally estimated using the spectral data (S_1_, CTS) in analogy to previous literature^22^, or based on the phosphorescence data^32^ for similar Bodipy compounds (T_1_)

The structures of the targeted compounds **T-1** and **T-2** for synthesis are shown in Fig. 2a. Terpyridyl-Bodipy compounds **T-1** and **T-2** differ only in the groups attached to the boron bridge. While compound **T-2** is appropriate for chemical characterization, compound **T-1** is better for biological media due to additional solubilizing oligoethyleneglycol units. The use of terpyridine instead of bipyridine ligand is favored due to the stronger affinity of the former ligand to Zn^2+^ ions in aqueous media. The changes in emission were studied in acetonitrile. A 2.0 μM solution of the model DEMUX **T-2** has an intense emission band (*ϕ*_F_ = 0.34) with a maximum at 517 nm. Titration with Zn^2+^ ions in the form of perchlorate salt results in sharp loss of emission intensity, with emission band moving to longer wavelengths, as the CT component in the emission becomes more prominent.

**Fig. 3:**
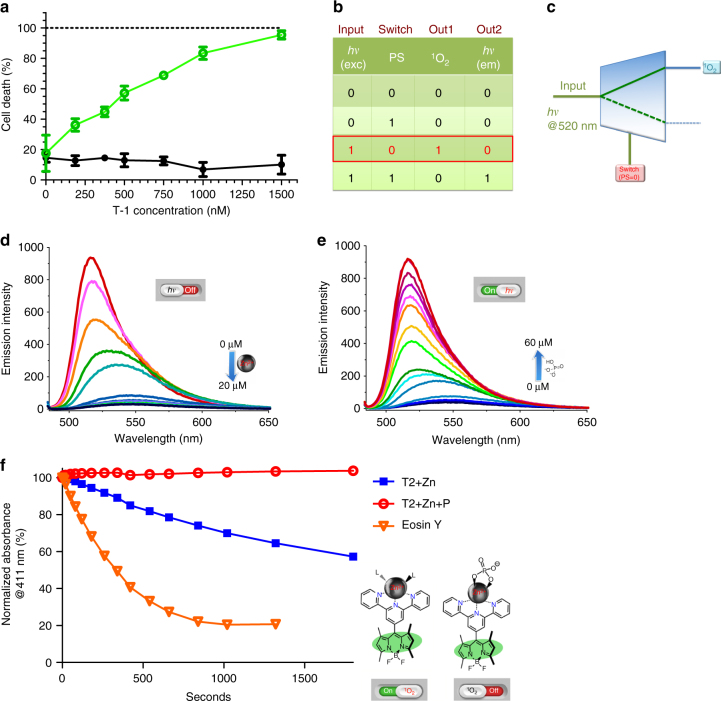
Operation of the molecular automata as evidenced by spectroscopic and cell culture data. **a** MTT assay data: Green open circles correspond to percent death of K562 cells under irradiation at 522 nm for 12 h followed by continued incubation in dark for another 12 h. Solid black circles correspond to cells kept under identical conditions of incubation with the agent, but in dark. Positive control (dashes at 100% line) corresponds to cells incubated in DMSO-growth medium mixture (50/50, *v*/*v*). **b** The truth table with the data and switch inputs, and the corresponding outputs clearly identified, and the particular set of conditions valid under the light irradiation conditions on power-up were highlighted. **c** Switch input 0 (no added phosphate in the model system or lack of phosphatidylserine (PS) in the external leaflet of the cell membrane in the cell cultures) selects singlet oxygen as the primary output. **d** The model compound **T-2**, which is the Zn^2+^ complex of the *meso*-terpyridyl-bodipy compound, has a very low fluorescence emission intensity in acetonitrile. **e** The addition of phosphate ions results in a very sharp increase in emission intensity. The low emission intensity is due to the availability of a charge transfer state (CTS) resulting in enhanced intersystem crossing, which in turn leads to efficient generation of singlet oxygen. **f** The decrease in the absorbance at 411 nm, in an oxygen saturated ethanol solution of selective singlet oxygen trap DPBF (50 µM) in the presence of 2.0 µM **T-2** and under irradiation with 522 nm green LED light source (solid blue squares). The singlet oxygen quantum yield (*ϕ*_Δ_) of **T-2** is 0.11. The addition of phosphate (red open circles) destabilizes the CTS state, blocking access to the triplet manifold. The absorbance data presented is the net absorbance values obtained by subtracting any background decrease in the probe absorbance due to light alone

However, the addition of tetrabutylammonium phosphate completely reverses this change at 60 μM concentration in acetonitrile. We then studied singlet oxygen generation rates under the same conditions for +/– phosphate: the singlet oxygen trap used is 1,3-diphenylisobenzofuran, its absorbance at 410 nm decreases due to a [4+2] cycloaddition with singlet oxygen, which is followed by decomposition. In accordance with our design expectations, when irradiated with a *λ* = 522 nm light-emitting diode array (at 98.0 µmol m^−2^ s^−1^ photon flux), the Zinc^2+^ complex is quite an effective photosensitizer. The singlet oxygen quantum yield (*ϕ*_Δ_) was determined in reference to Eosin Y, and found to be 0.11. However, the addition of 2.0 mM phosphate in the form of tetrabutylammonium phosphate essentially stops singlet oxygen generation (Fig. 3f). Thus, it is clear that fluorescence emission intensity and the singlet oxygen generation efficiency are inversely coupled, and the switch is the phosphate ions in solution. Operation of the molecular DEMUX circuit based on compound **T-2** was confirmed. We also determined the singlet oxygen quantum yield of **T-1** as 0.10.

Once the chemical validation was obtained, we ventured into cell culture experiments. Cell culture assays were performed with a human cancer suspension cell line-chronic myelogenous leukemia (K562). The cells were incubated with Dulbecco’s modified Eagle's medium (DMEM) supplemented with 20% fetal bovine serum at the environmental conditions of 37° C, 5% CO_2_, and 60% humidity. Cells were treated with varying concentrations of the molecular automaton **T-1** (187.5 nM–1.5 µM) and illuminated with a green light source (*λ* = 522 nm light-emitting diode array, 98.0 µmol m^−2^ s^−1^ photon flux) for a continuous duration of 12 h. This 12 h period of illumination was followed by 12 h of incubation in the dark (total 24 h). The control group of the cells were incubated in the dark, for the exact duration of 24 h under identical environmental conditions. The MTT (3-(4, 5-dimethylthiazolyl-2)−2,5-diphenyltetrazolium bromide) assay was used in order to assess cell viability and cytotoxicity. Even the low doses of the automaton **T-1** seem to have resulted in a significant decrease of the cell viability (Fig. 3a, points and error bars designate means and standard deviations, respectively). The CC_50_ (50% cytotoxic concentration) value of the **T-1** subjected to green light was estimated by fitting a model with non-linear regression (approximately 365 nM; the CC_50_ value of the compound in the dark condition cannot be estimated since its projection is clearly out of the scale of the model fit).

### Flow cytometry and microscopy

**Fig. 4:**
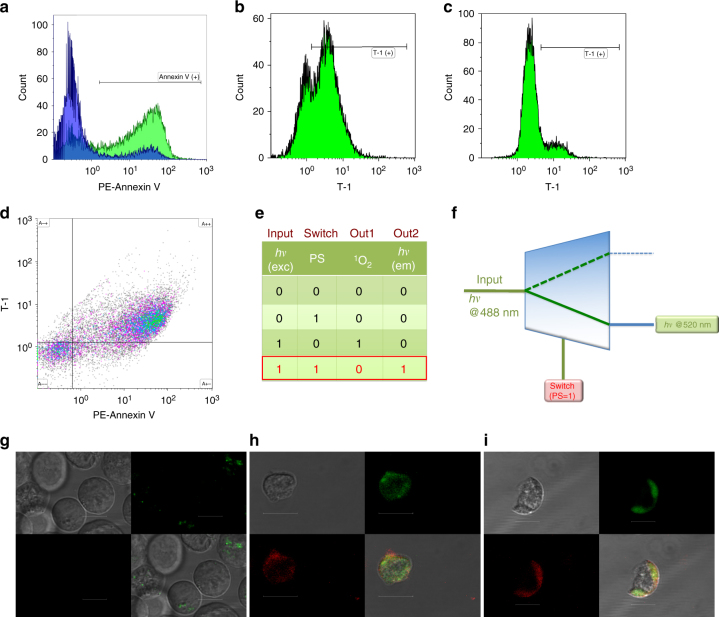
**T-1** signals apoptosis by switching to diagnostic mode. **a** PE-Annexin labels most of the cells incubated with **T-1** under light irradiation (Annexin V (+) region of the green area). **b** Green channel: cells incubated with **T-1** under irradiation. **c** Green channel: cells incubated with **T-1** in the dark. **d** The 2D plot for both green and red channels: **T-1** and PE-Annexin V (a specific apoptosis marker) stain the same kind of cells with a large (86%) agreement: 70% of the irradiated cells were co-stained with both **T-1** and PE-Annexin V, indicating only apoptotic cells are fluorescently labeled with **T-1**. **e** The truth table with input, the switch and the outputs clearly identified, and the particular set of conditions were highlighted. **f** Switch input (appearance of phosphatidylserine in the external leaflet of the cell membrane) selects fluorescence emission as the primary output. **g** Cells treated with PE-Annexin V and the **T-1**, and kept in dark, show no signs of morphological change, and the cellular membranes are not stained with either one of the agents. **h**, **i** Cells were incubated with **T-1**, irradiated with the LED light source, then treated with PE-Annexin V. The two agents (**T-1** and PE-Annexin V, green and red, respectively) label the same regions in the cells undergoing apoptosis. (scale bar, 10 μm)

In order to confirm the MTT assay results, and the switch from singlet oxygen generation to the emission/signaling mode; Annexin V detection protocol for apoptosis was performed using flow cytometry. The percentage of fluorescent-labeled Annexin V (phycoerythrin (PE))-stained cells was much higher in the illuminated cell population compared with those incubated in the dark (73.7 vs. 18.0%; Fig. 4a, blue shaded areas represent the cells in the dark, green shaded areas represent the irradiated cells). In addition, 71.7% of irradiated K562 cells (Fig. 4b) were positive for **T-1** in contrast to 18.0% of the cells incubated in the dark (Fig. 4c), demonstrating that Annexin V and the automaton targets the same kind of cell membranes with outer leaflet enriched in phosphatidylserine, which are undergoing apoptosis. Cancer cells, which are not killed, or not undergoing apoptosis, are not marked by the agent, but subjected to cytotoxic singlet oxygen attack, when the automaton is powered up by irradiation. Photocytotoxicity was further revealed in a 2-color analysis with PE-Annexin V in order to specifically target and identify apoptotic cells in conjunction with the **T-1** (Fig. 4d). Approximately 70% of the illuminated cells were positive for both PE-Annexin V and **T-1** and about 16% of the cells were negative for both (area A++ and A−− in Fig. 4d, respectively), confirming that the terminator automaton turns on the emission signal only when the cells are undergoing apoptotic death process.

### Microscopy

Confocal microscopy provided further evidence corroborating the cytotoxicity results from MTT assays and flow cytometry, as well as the co-staining of PE-Annexin V and **T-1** in flow cytometry analyses. The data demonstrate that just like PE-Annexin V, the automaton shows fluorescence signal only when attached to apoptotic cell membranes. Cellular membranes of the illuminated cells incubated with **T-1** were shown to be stained with both Annexin V and **T-1** (1.5 µM); in contrast to their counterparts incubated in the dark, which were not stained by either** T-1** (1.5 µM) or PE-Annexin V. Cell membranes of the illuminated cells in the presence of **T-1** appear bright green when excited at 488 nm and bright red when excited at 543 nm. However, their counterparts, which had been incubated in the dark, were negative for either green or red emission. Figure 4g–i shows the differential interference contrast (DIC) image, **T-1**, Annexin V and merged images, respectively.

**Fig. 5:**
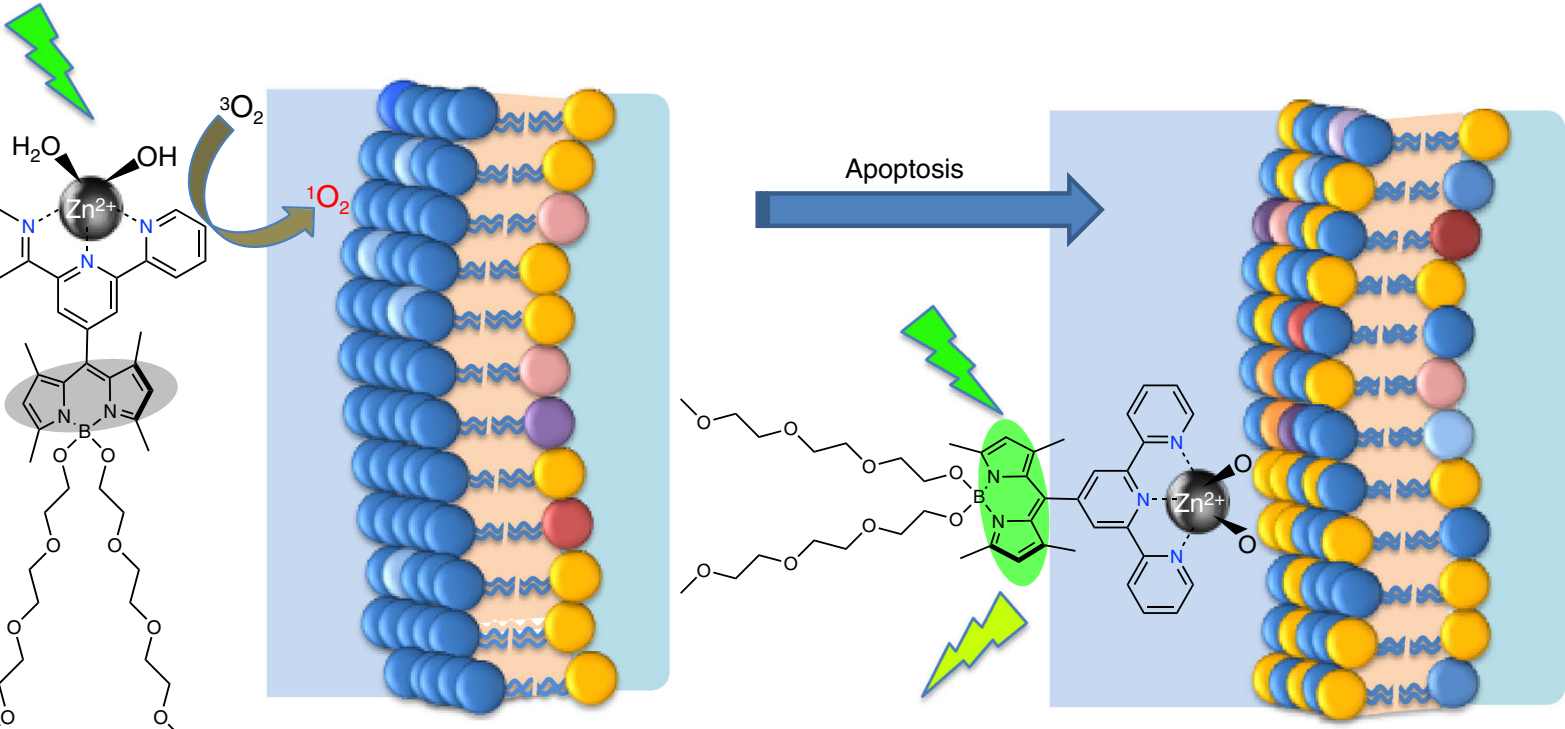
**T-1** induces apoptosis and then switches to diagnostic mode and fluorescently tags apoptotic cells. Blue polar heads represent phosphatidylcholine and sphingomyelins, whereas yellow, pink and purple heads represent phosphatidylserine, phosphatidylinositol and other negatively charged lipids. Apoptosis is accompanied by a loss of membrane asymmetry

A graphical representation of the operation of the automaton **T-1** is shown in Fig. 5.

## Discussion

It appears that judiciously designed molecular logic devices can carry out critical information processing in or around the cells. Widely speculated nanorobots are not expected to be miniaturized versions of their macroscopic counterparts, but as illustrated here, most likely to be developed by careful control of photophysics and chemical reactivity at the individual molecule level, resulting in molecular entities with intelligent responses and actions. The zinc complex of the terpyridyl-bodipy, **T-1**, is approximately 3 nm at the longest dimension, yet it is capable of killing cancer cells when powered up by light, and then autonomously switching to a signaling mode in response to changing membrane characteristics, thus confirming apoptosis by a strong emission signal. As evidenced by the spectroscopic experiments with **T-2**, and flow cytometry data obtained with **T-1**, emission intensity and the singlet oxygen production is inversely coupled, and the fact that singlet oxygen generation is turned off once the cells show signs of apoptosis is very valuable in containing unintended damage by singlet oxygen. This clearly qualifies **T-1** as a molecular terminator automaton, or an example of a functional molecular robot. While most nano-sized robotics work is at present focussed on mobility, it is interesting to note that mobility is far from being the number one issue for a functional, molecule-sized robot. Most infectious microorganisms, cells fighting against these agents, and drug molecules are efficiently carried passively via the circulatory system to any part of the body. It is the intelligent autonomous operation that provides the most crucial challenge, and the terminator automaton **T-1** demonstrates one way of moving ahead to meet that challenge.

## Methods

### Cell culture and MTT assay

K562 human chronic myelogenous leukemia suspension cells were cultured in 25 cm^2^ culture flasks containing DMEM (Gibco, 11971-025) supplemented with 20% fetal bovine serum in a cell culture incubator at 37 °C, 5% CO_2_, and 60% humidity. The main functional goals of the current study are to study the cytotoxicity of **T-1** under illumination and its staining ability of the apoptotic cells. Since phosphate in the growth media can interfere with experimental results, we used DMEM w/o phosphate supplemented with 20% fetal bovine serum that has been dialyzed extensively at 4 °C against isotonic saline (0.15 M NaCl) using dialysis tubing (Sigma-Aldrich, USA (D7884)), since the viability of the cells cultured in such conditions is known to be not hampered for periods even more than 24 h^28^. This cell culture medium was also utilized for analyses with flow cytometry and confocal laser scanning microscopy to study cytotoxicity and cellular staining.

The compound was diluted in cell culture medium and assay concentrations were freshly prepared. Cell viability/death was evaluated by MTT assay. Briefly, 50 µl cell suspensions in culture medium containing 3 × 10^4^ K562 cells were plated in 96-well flat-bottom culture plates (Corning, MA, USA) and incubated for 12 h to recover from handling. Varying concentrations of the chemical compound in cell culture medium were added into each well (the final concentrations were 187.5 nM–1.5 µM) in quadruplicate. The experimental group of the cells were illuminated with a green light source (*λ* = 522 nm light-emitting diode array, 98 µmol m^−2^ s^−1^ photon flux, distance between light source and cells: 10 cm) for a continuous duration of exactly 12 h in a culture incubator (37 °C, 5% CO_2_, 60% humidity). This 12 h period of illumination was followed by 12 h of incubation solely in the dark (total 24 h) also in the incubator. The control group of the cells were incubated in the dark, for the exact duration of 24 h under identical environmental conditions except illumination. According to the assay protocol, 25 µl of the MTT reagent (Sigma-Aldrich, MO, USA) was added to each well in order to assess cell viability (final concentration: 1 mg ml^−1^) at the end of the 24 h of incubation period. Following 4 h of incubation of the cells with the MTT reagent, the generated formazan precipitates were solubilized by the addition of the lysing buffer (80 µl, pH: 4.7), which is composed of 23% sodium dodecyl sulfate (SDS) dissolved in a solution of 45% *N*,*N*-Dimethylformamide (DMF). After an overnight incubation at 37 °C, the absorbance values (of each well) were measured at 570 nm in a microtiter plate reader (Spectramax Plus, Molecular Devices, CA, USA) at 25 °C. Cells incubated in culture medium only (without the compound) served as the control for cell viability both for the illuminated plates and for the ones kept in the dark; whereas dimethyl sulfoxide (DMSO; 50%, *v*/*v*) was used to observe maximum cell death (positive control). Cell death (%) was assessed with the normalization of the values calculated by the formula 'optical density (OD) of control cells − OD of treated cells'. The CC_50_ values of the compound under illumination conditions were estimated by fitting a model with non-linear regression.

### Flow cytometry

Illuminated and control K562 cells (in the dark) were stained with PE-Annexin V, as described in the technical data sheet (BioLegend, USA), and were analyzed by FACS Aria II (equipped with 488 nm and 635 nm lasers) using FACS Diva software.

### Confocal laser scanning microscopy

Illuminated and control (dark) K562 cells were analyzed under a confocal laser scanning microscope (Zeiss LSM 510 META, Germany) at the excitation wavelengths of 488 or 543 nm to view the green (**T-1**) and red (PE-Annexin V) fluorescence, respectively, in order to investigate the correlation of staining of the cells with **T-1** and Annexin V; operating in the sequential (multitrack) excitation/recording mode to eliminate a possible cross-talk between the channels with recording the fluorescence signal in the green (BP 505–530 nm) or red (LP 560) channel. Annexin V serves as a sensitive marker for detection of cells that are undergoing apoptosis. Images were captured at a magnification of ×63, 1.4 numerical aperture objective and a scan speed of 400 Hz.

### Synthesis of **T-2**

TFA (0.22 ml, 2.87 mmol) was added dropwise to a vigorously stirring solution of 4′-formyl-2,2′:6′,2”-Terpyridine^29,30^ (0.5 g, 1.91 mmol) and 2,4-dimethylpyrrole (0.473 ml, 4.58 mmol) in 500.0 ml argon deaerated dichloromethane (DCM). The resulting solution was left to stir at room temperature in the dark for 1 day. *p-*Chloranil (0.47 g, 1.91 mmol) was added in one portion and reaction was left to stir for 2 h. Diisopropylethylamine (8.0 ml) was added dropwise to this mixture over a period of 15 min, and the resulting dark brown solution was allowed to stir for an additional 30 min. BF_3_.OEt_2_ (8.0 ml) was then added dropwise over a period of 15 min and the resulting dark red solution was allowed to stir at room temperature in the dark for 1 day. The slurry reaction mixture was washed with water (3 × 300 ml) and dried over anhydrous Na_2_SO_4_. The solvent was evaporated and the residue was purified by using neutral Al_2_O_3_ using DCM: Hexane (1:1, *v*/*v*) as the eluent to afford compound **T-2** (0.37 g, 40.4%). ^1^H nuclear magnetic resonance (NMR) (400 MHz, CDCl_3_): δ_H_ 8.70–8.73 (m, 4H), 8.57 (s, 2H), 7.92 (td, *J = *1.9, 7.6 Hz, 2H), 7.37–7.40 (m, 2H), 6.01 (s, 2H), 2.60 (s, 6H), 1.56 (s,6H). ^13^C NMR (100 MHz, CDCl_3_) *δ* 156.4, 156.1, 155.3, 149.4, 145.3, 142.8, 138.8, 136.9, 130.5, 124.3, 121.6, 121.1, 120.6, 30.9, 15.2 ppm. High-resolution mass spectrometry (HRMS) (time-of-flight electrospray-ionization (TOF-ESI)): *m/z*: calculated: 479.22019, Found: 479.22179 [M + H]^+^, Δ = −3.21 ppm.

### Synthesis of **T-1**

A sample of **T-2** (0.05 g, 0.10 mmol) was dissolved in 3.0 ml DCM. Triethyleneglycol monomethyl ether (0.168 g, 1.0 mmol) was added to the reaction mixture which was stirred at 45 °C. The reaction was started with the addition of AlCl_3_ (0.031 g, 0.23 mmol). The progress of the reaction was followed by thin-layer chromatography (neutral Al_2_O_3_, DCM:MeOH [98:2, *v*/*v*]). When all the starting material was consumed, reaction medium was cooled down to room temperature and filtered. The filtrate was concentrated under vacuum and the residue was purified by using neutral Al_2_O_3_ using DCM: MeOH (98:2, *v*/*v*) as the eluent to afford compound** T-1** (0.068 g, 88.3%). ^1^H NMR (400 MHz, CDCl_3_): δ_H_ 8.75–8.70 (m, 4H), 8.54 (s, 2H), 7.95–7.90 (m, 2H), 7.41–7.37 (m, 2H), 5.95 (s, 2H), 3.66–3.54 (m, 2H), 3.39 (s, 6H), 2.59 (s, 6H), 1.52 (s, 6H). ^13^C NMR (100 MHz, CDCl_3_) *δ* 156.6, 156.2, 155.4, 149.3, 146.1, 144.5, 141.1, 140.7, 138.3, 137.0, 131.2, 124.3, 121.5, 121.2, 120.8, 96.4, 73.1, 72.3, 71.9, 70.7, 70.5, 70.4, 60.7, 59.0, 15.3, 14.9. HRMS (TOF- ESI): *m/z*: calculated: 789.39940, Found: 789.40056 [M + H]^+^, Δ = −1.47 ppm.

### Data availability

The authors declare that the data supporting the findings of this study are available within the article and its Supplementary Information files.

## Electronic supplementary material

Supplementary Information
