# Supplementary material for: Molecular demultiplexer as a terminator automaton
Source: Nat Commun. 2018 Feb 23;9:805. doi: 10.1038/s41467-018-03259-z (PMC5824880; doi:10.1038/s41467-018-03259-z)
Supplement: Supplementary file 1 — Supplementary Information [file 41467_2018_3259_MOESM1_ESM.pdf]

Supplementary Information for

Molecular demultiplexer as a terminator automaton

Ilke S. Turan, Gurcan Gunaydin, Seylan Ayan, Engin U. Akkaya*.

*correspondence to: eua@fen.bilkent.edu.tr

Supplementary Methods

General Experimental Procedures

^{1}H NMR and ^{13}C NMR spectra were recorded on Bruker Spectrospin Avance DPX 400 spectrometer using $CDCl_3$ as the solvent. Chemical shifts values are reported in ppm from tetramethylsilane as internal standard. Spin multiplicities are reported as the following: s (singlet), d (doublet), m (multiplet). Electronic absorption spectra in solution were acquired using a Varian Cary-100 spectrophotometer and a StellarNet BLACK Comet C-SR diode array miniature spectrophotometer connected to deuterium and halogen lamp by optical fiber using 1 cm matched quartz cuvettes at room temperature. Fluorescence spectra were determined on Varian Eclipse and Edinburgh Instruments FLS920 fluorospectrometer. Spectrophotometric grade solvents were used for spectroscopy experiments. Fluorescence lifetime of S1 state was measured by time-correlated single photon counting method (Edinburgh FLS920 spectrophotometer) with excitation at 507 nm by a portable diode laser (150 ps FWHM) and emission was monitored at 558 nm. The lifetime values were computed by the F900 software. All spectra were corrected for the sensitivity of the photo-multiplier tube. HRMS data were acquired on an Agilent Technologies 6530 Accurate-Mass Q-TOF LC/MS. Flash column chromatography (FCC) was performed by using glass columns with a flash grade silica gel (Merck Silica Gel 60 (40–63 µm)). Reactions were monitored by thin layer

chromatography (TLC) using precoated silica gel plates (Merck Silica Gel PF-254), visualized by UV-Vis light. All organic extracts were dehydrated over anhydrous Na_2SO_4 and concentrated by using rotary evaporator before being subjected to FCC. In singlet oxygen measurements 1,3-Diphenylisobenzofuran was usedas a singlet oxygen trap in organic solvent measurements and was purchased from supplier. All other chemicals and solvents were supplied from commercial sources and used as received. Reference compound was synthesized based on the literature[30]. Titrations were performed by using ZnOAc as Zn source and tetrabutylammonium phosphate as phosphate source. ZnOAc is represented as Zn and phosphate source is represented as P in the graphs.

Synthetic Procedures

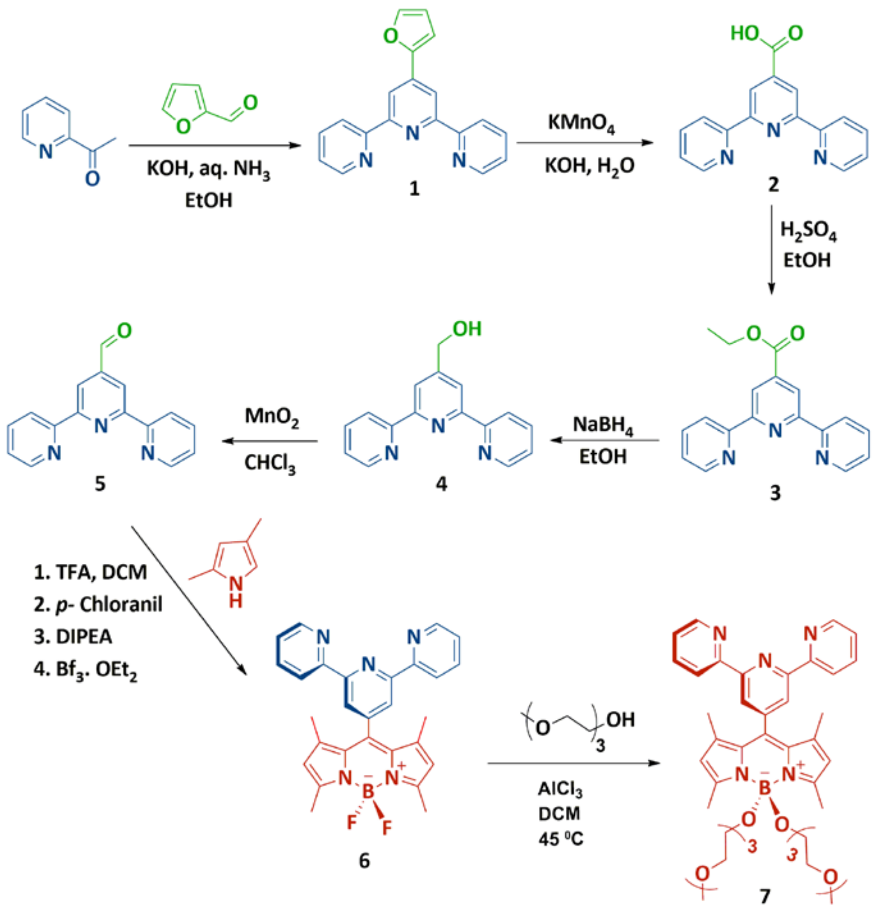

Supplementary Figure 1. Total synthesis of **T-1** (**7**).

Synthesis of 4'-(2-furanyl)-2,2':6',2''-Terpyridine (**1**):

Furfuraldehyde (1.92 g, 20.0 mol) was added to the reaction mixture containing 2-acetylpyridine (4.48 g, 40.0 mmol) dissolved in ethanol (90.0 ml). KOH (3.08 g, 54.5 mmol) and 25% (wt/vol) aq. NH_3 (58.0 ml, 1.51 mmol) were added to reaction mixture which was left to stir at room temperature for 24 hours. Solids were filtered under

vacuum onto a sintered funnel and washed with ice cold 50% (*v*/*v*) ethanol until the washings become colorless. Then, the solid was dried to obtain the compound **1** (2.24 g,56%).

^{1}H NMR (400 MHz, *d6*-DMSO): δ_H 8.76 (d, *J* = 4.8 Hz, 2H), 8.69 (s, 2H), 8.65 (d, *J*= 7.9 Hz, 2H), 8.04 (t, *J*= 7.7 Hz, 2H), 7.97 (s, 1H), 7.54 (dd, J= 4.8, 7.4 Hz, 2H),7.46 (d, J= 3.4 Hz, 1H), 6.75 (s, 1H).

^{13}C NMR (100 MHz, *d6*-DMSO) δ 156.2, 155.3, 151.2, 149.8, 145.5, 139.5, 137.9, 125.1, 121.3, 114.6, 113.2, 110.8 ppm.

MS (TOF- ESI): m/z: calculated: 300.11314, Found: 300.11265 $[M+H]^+$, Δ= 1.56 ppm.

Synthesis of [2,2':6',2''-Terpyridine]-4'-carboxylic acid (**2**):

KOH pellets were added to demineralized water (120.0 ml) until the pH of the solution is adjusted to 10. Then, furyl-terpyridine **1** (2.24 g, 7.48 mmol) was added to the reaction mixture. After the addition of KMnO4 (4.72 g, 29.87 mmol), reaction mixture was refluxed for 3 hours. After cooling the reaction medium to room temperature, reaction mixture was filtered through a pad of Celite which deposited on a sintered funnel and washed with demineralized water. The pH of the filtrate was adjusted to pH=5 by dropwise addition of concentrated HCl to afford white precipitate which was filtered via

glass-sintered funnel and wash thoroughly with demineralized water till the washings become neutral. Then, the solid was dried to obtain the compound **2** (1.36 g, 66%).

^{1}H NMR (400 MHz, CH_3OD): δ_H 8.95 (s, 2H), 8.73 (d, *J* = 4.8 Hz, 2H), 8.68 (d, *J*= 7.9 Hz, 2H), 8.04 (td, *J*= 1.7, 7.8 Hz, 2H).

^{13}C NMR (100 MHz, CH_3OD) δ 156.4, 155.2, 148.9, 137.5, 124.4, 121.4, 120.1, 111.9 ppm.

MS (TOF- ESI): m/z: calculated: 278.0924, Found: 278.09255 $[M+ H]^+$, Δ= -0.53 ppm.

Synthesis of ethyl- [2,2':6',2''-Terpyridine]-4'-carboxylate (**3**):

[2,2':6',2''-Terpyridine]-4'-carboxylic acid 2 (1.36 g, 4.90 mmol) was dissolved in absolute ethanol (150.0 ml). After the addition of concentrated H_2SO_4 (1.0 ml), reaction mixture was refluxed for 72 hours. After the removal of ethanol under vacuum, the residue was dissolved in water (100.0 ml) and the pH of the solution was adjusted to 8.0 by portionwise addition of solid $NaHCO_3$. Aqueous layer was extracted with dichloromethane, dried over Na_2SO_4 and concentrated under vacuum to isolate the compound **3** (1.20 g, 87%).

^{1}H NMR (400 MHz, $CDCl_3$): δ_H 9.02 (s, 2H), 8.78 (s, 2H), 8.65 (d, *J*= 7.9 Hz, 2H), 7.91 (t, *J*= 7.7 Hz, 2H),7.38-7.41 (m, 2H), 4.51 (q, *J*= 14.3 Hz, 2H), 1.49 (t, *J*= 7.1 Hz, 3H).

^{13}C NMR (100 MHz, $CDCl_3$) δ 156.6, 149.3, 16.9, 126.9, 124.1, 121.3, 120.4, 61.8, 14.4 ppm.

MS (TOF- ESI): m/z: calculated: 306.12370, Found: 306.12428 $[M+H]^+$, Δ= -1.95 ppm.

Synthesis of [2,2':6',2''-Terpyridine]-4'-methanol (**4**):

Ethyl- [2,2':6',2''-Terpyridine]-4'-carboxylate (1.0 g, 3.28 mmol) was dissolved in ethanol (100.0 ml). After the addition of $NaBH_4$ (2.48 g, 65.6 mmol), reaction mixture was refluxed at 85°C. The progress of the reaction was followed by TLC (neutral Al_2O_3, DCM). When all the starting material was consumed, reaction medium was cooled to room temperature. Saturated $NaHCO_3$ solution was added to the reaction mixture in order to deactivate excess $NaBH_4$. After the removal of ethanol under vacuum, the residue was dissolved in water and extracted with ethyl acetate, dried over Na_2SO_4 and concentrated under vacuum to isolate the compound **4** (0.86 g, quantitative)[31].

^{1}H NMR (400 MHz, $CDCl_3$): δ_H 8.69 (s, 2H), 8.64 (d, *J*= 7.8 Hz, 2H), 8.41 (s, 2H), 8.02 (t, *J*= 7.7 Hz, 2H), 7.49 (t, *J*= 6.2 Hz, 2H), 4.86 (s, 2H).

^{13}C NMR (100 MHz, $CDCl_3$) δ 156.2, 155.6, 152.3, 149.1, 136.9, 123.8, 121.4, 118.5, 63.9 ppm.

MS (TOF- ESI): m/z: calculated: 264.11314, Found: 264.11434 $[M+H]^+$, Δ=-4.55 ppm.

Synthesis of 4'-formyl-2,2':6',2''-Terpyridine (**5**):

[2,2':6',2''-Terpyridine]-4'-methanol (0.86 g, 3.27 mmol) was dissolved in $CHCl_3$. After the addition of MnO_2 (2.84 g, 32.7 mmol), the reaction mixture was refluxed until all the starting material was consumed. The progress of the reaction was followed by TLC (neutral Al_2O_3, DCM). When all the starting material was consumed, reaction medium was cooled to room temperature and filtered. The filtrate was concentrated under vacuum to isolate the compound **5** (0.85 g, quantitative).

^1H NMR (400 MHz, $CDCl_3$): δ_H 10.31 (s, 1H), 8.92 (s, 2H), 8.78 (s, 2H), 8.66 (d, *J*= 8.2 Hz, 2H), 7.93 (t, *J*= 7.8 Hz, 2H), 7.42 (t, *J*= 6.1 Hz, 2H).

^{13}C NMR (100 MHz, $CDCl_3$) δ 191.7, 157.1, 155.0, 149.3, 143.9, 136.9, 124.3, 121.2, 119.7 ppm.

MS (TOF- ESI): m/z: calculated: 262.09749, Found: 262.09824 $[M+H]^+$, Δ= -2.87 ppm.

Synthesis of 4,4-difluoro-1,3,5,7-tetramethyl-8-(2,2':6',2''-Terpyridine)-4-bora-3a,4a-diaza-s-indacene(**6**):

Trifluoroacetic acid (TFA; 0.22 ml, 2.87 mmol) was added dropwise to a vigorously stirring solution of 4'-formyl-2,2':6',2''-Terpyridine (0.5 g, 1.91 mmol) and 2,4-dimethylpyrrole (0.473 ml, 4.58 mmol) in 500.0 mlargon deaerated dichloromethane (DCM). The resulting solution was left to stir at room temperature in the dark 1 day. *p*-Chloranil (0.47 g, 1.91 mmol) was added in one portion and reaction was left to stir for 2 hours. Diisopropylethylamine (8.0 ml) was added dropwise to this mixture over a period of 15 min, and the resulting dark brown solution was allowed to stir for an additional 30.0 min. $BF_3.OEt_2$ (8.0 ml) was then added dropwise over a period of 15 min and the resulting dark red solution was allowed to stir at room temperature in the dark for 1 day. The slurry reaction mixture was washed with water (3 × 300 ml) and dried over anhydrous Na_2SO_4. The solvent was evaporated and the residue was purified by using neutral Al_2O_3 using DCM: Hexane (1:1, *v*/*v*) as the eluent to afford compound **6** (0.37 g, 40.4%).

^{1}H NMR (400 MHz, $CDCl_3$): δ_H 8.70-8.73 (m, 4H), 8.57 (s, 2H), 7.92 (td, *J*= 1.9, 7.6 Hz, 2H), 7.37-7.40 (m, 2H), 6.01 (s, 2H), 2.60 (s, 6H), 1.56 (s,6H).

^{13}C NMR (100 MHz, $CDCl_3$) δ 156.4, 156.1, 155.3, 149.4, 145.3, 142.8, 138.8, 136.9, 130.5, 124.3, 121.6, 121.1, 120.6, 30.9, 15.2 ppm.

MS (TOF- ESI): m/z: calculated: 479.22019, Found: 479.22179 $[M+H]^+$, Δ= -3.21 ppm.

Synthesis of 4,4-bis(2-(2-(2-methoxyethoxy)ethoxy)ethoxy)-1,3,5,7-tetramethyl-8-(2,2’:6’,2’’-Terpyridine)-4-bora-3a,4a-diaza-s-indacene (**7**):

Bodipy ***6*** (0.05 g, 0.10 mmol) was dissolved in 3.0 ml DCM. Triethyleneglycol monomethyl ether (0.168 g, 1.0 mmol) was added to the reaction mixture which was stirred at 45 °C. The reaction was started with the addition of $AlCl_3$ (0.031 g, 0.23 mmol). The progress of the reaction was followed by TLC (neutral Al_2O_3,DCM:MeOH [98:2, *v*/*v*]). When all the starting material was consumed, reaction medium was cooled to room temperature and filtered. The filtrate was concentrated under vacuum and the residue was purified by using neutral Al_2O_3 using DCM: MeOH (98:2) as the eluent to afford compound ***7*** (0.068 g, 88.3%).

^{1}H NMR (400 MHz, $CDCl_3$): δ_H 8.75-8.70 (m, 4H), 8.54 (s, 2H), 7.95-7.90 (m, 2H), 7.41-7.37 (m, 2H), 5.95 (s, 2H), 3.66-3.54 (m, 24H), 3.39 (s, 6H), 2.59 (s, 6H), 1.52 (s, 6H).

^{13}C NMR (100 MHz, $CDCl_3$) δ 156.6, 156.2, 155.4, 149.3, 146.1, 144.5, 141.1, 140.7, 138.3, 137.0, 131.2, 124.3, 121.5, 121.2, 120.8, 96.4, 73.1, 72.3, 71.9, 70.7, 70.5, 70.4, 60.7, 59.0, 15.3, 14.9.

MS (TOF- ESI): m/z: calculated: 789.39940, Found: 789.40056 $[M+ H]^+$, Δ= -1.47 ppm.

Supplementary Figures

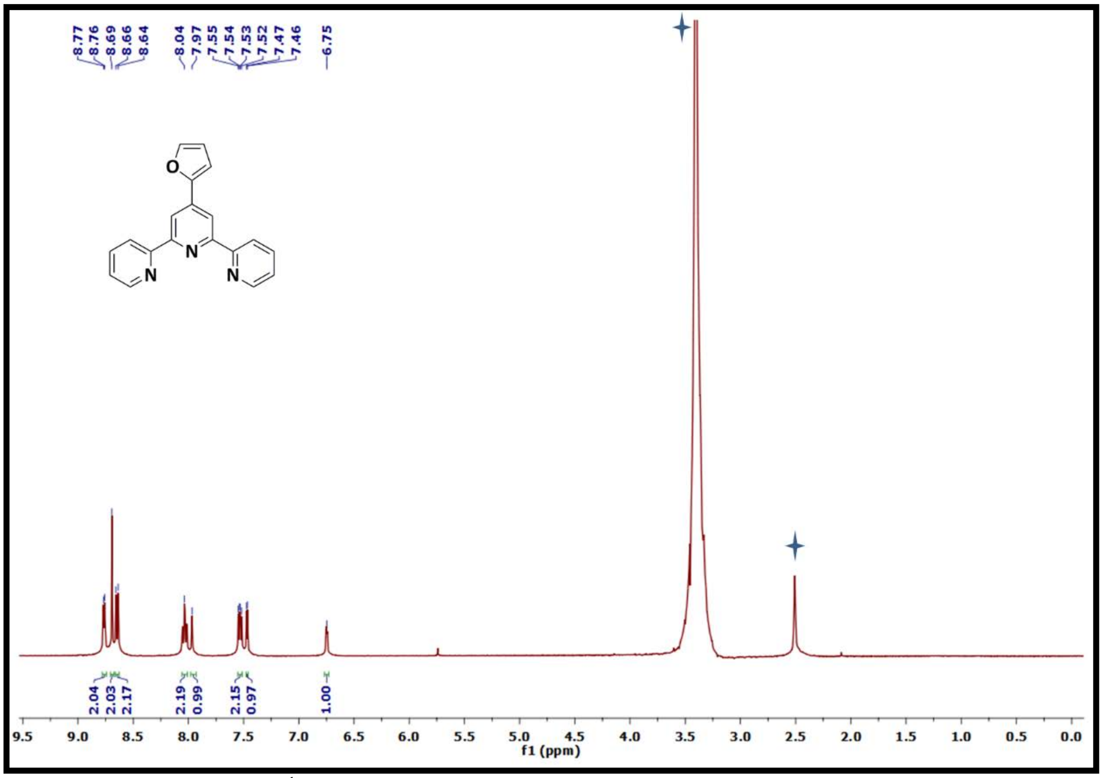

Supplementary Figure 2. ^{1}H-NMR Spectrum of Compound **1**.

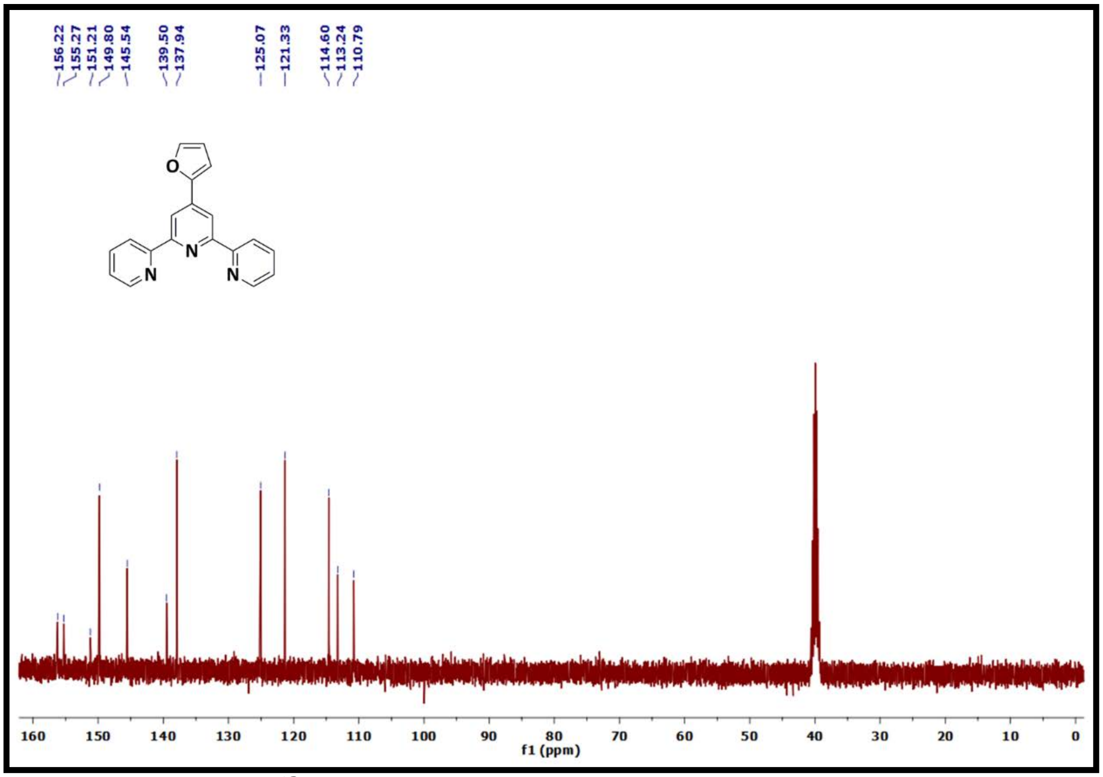

Supplementary Figure 3.^{13}C-NMR Spectrum of Compound **1**.

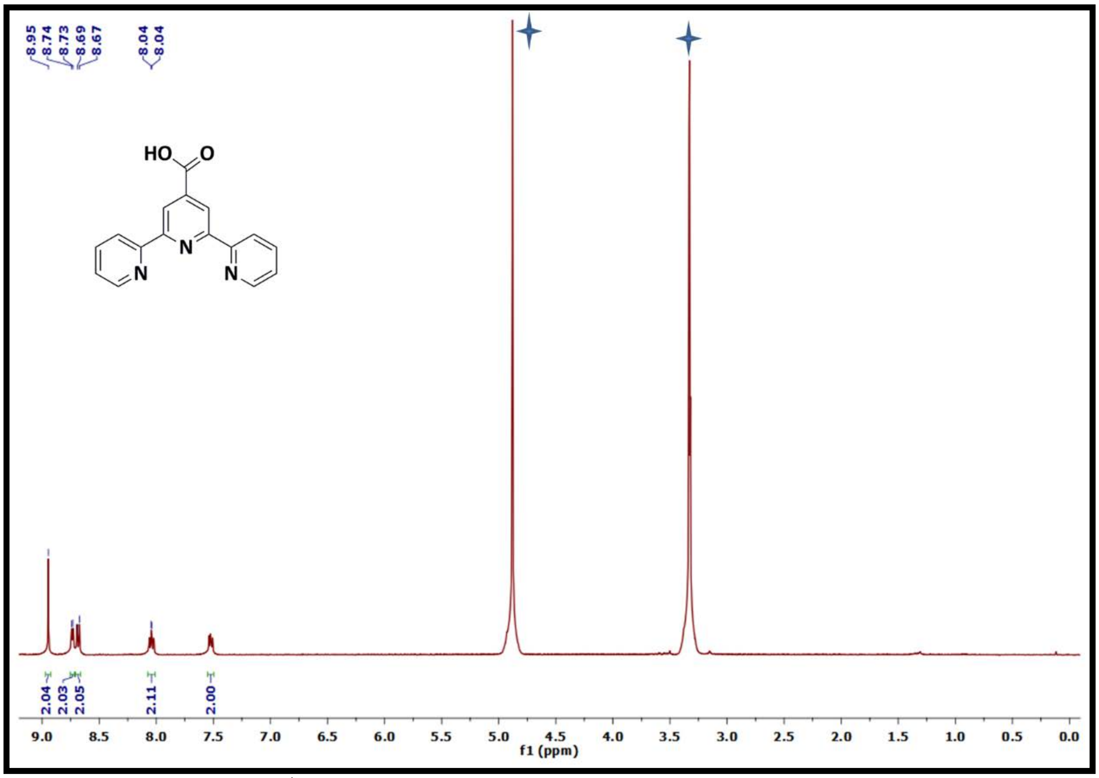

Supplementary Figure 4. ^{1}H-NMR Spectrum of Compound **2**.

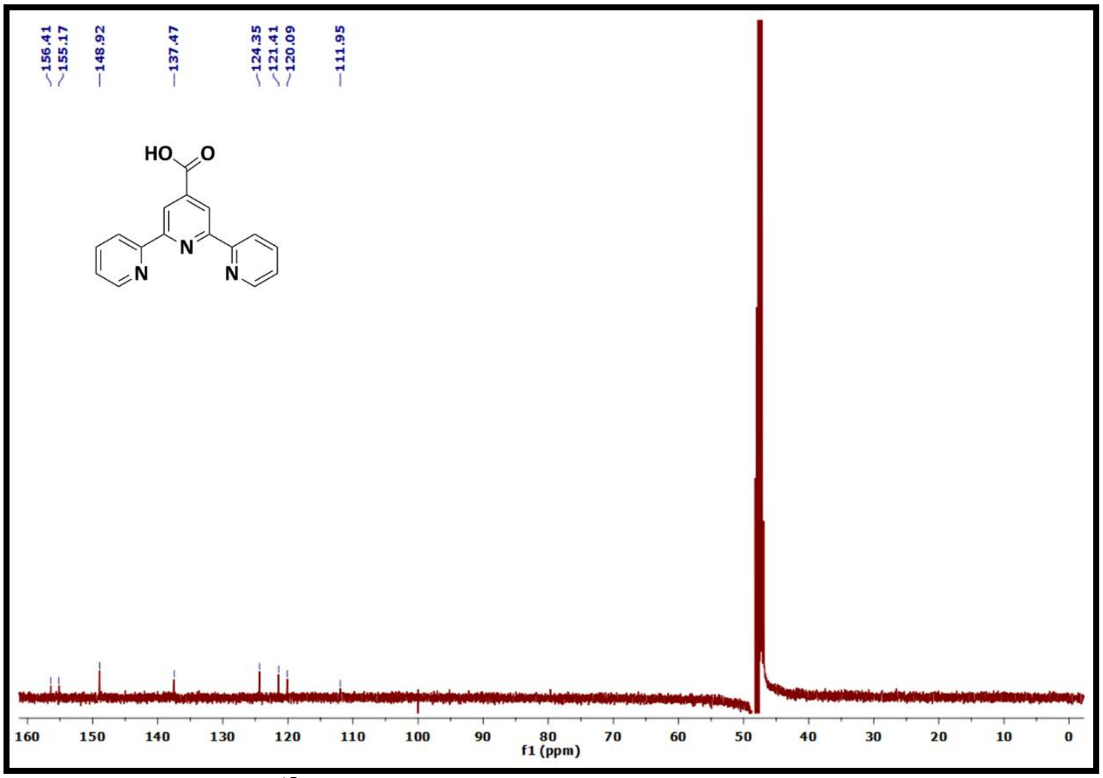

Supplementary Figure 5.^{13}C-NMR Spectrum of Compound **2**.

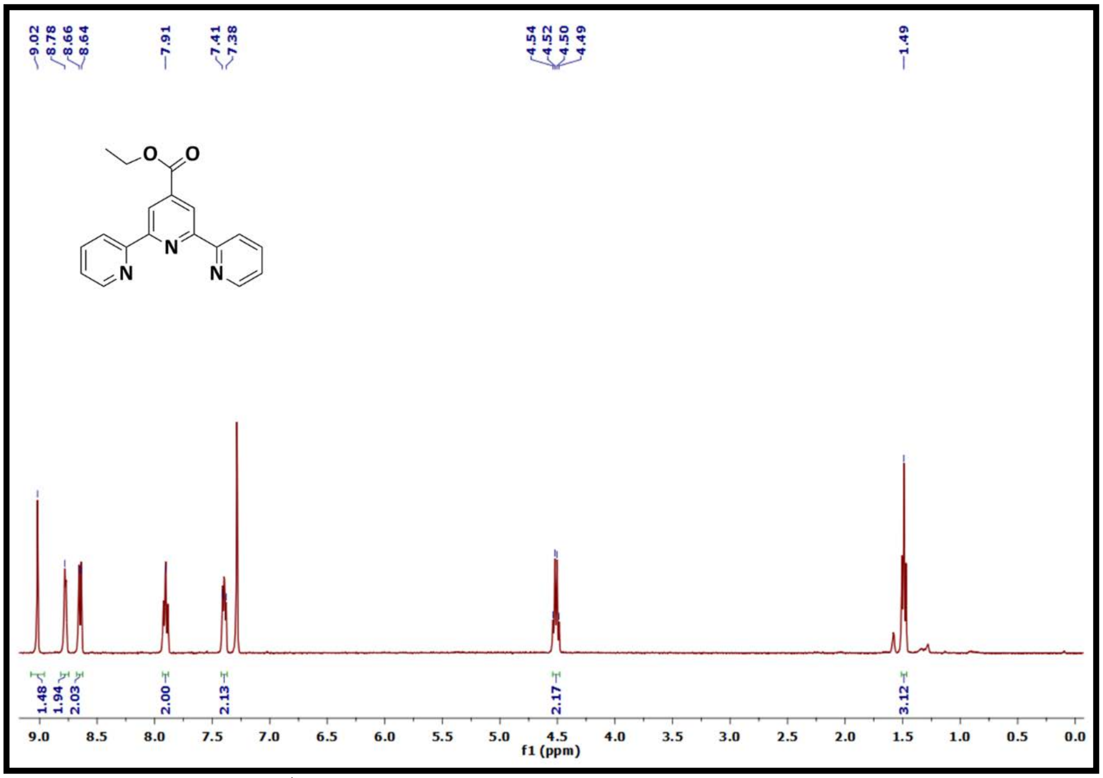

Supplementary Figure 6. ^{1}H-NMR Spectrum of Compound **3**.

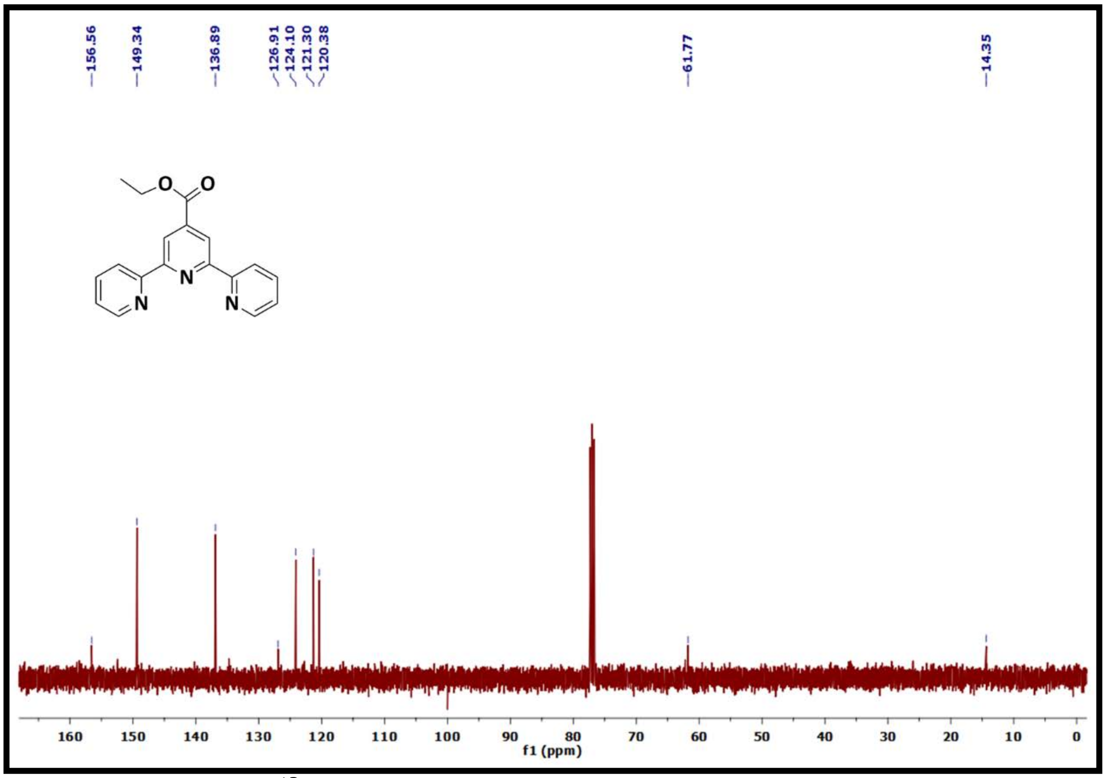

Supplementary Figure 7.^{13}C-NMR Spectrum of Compound **3**.

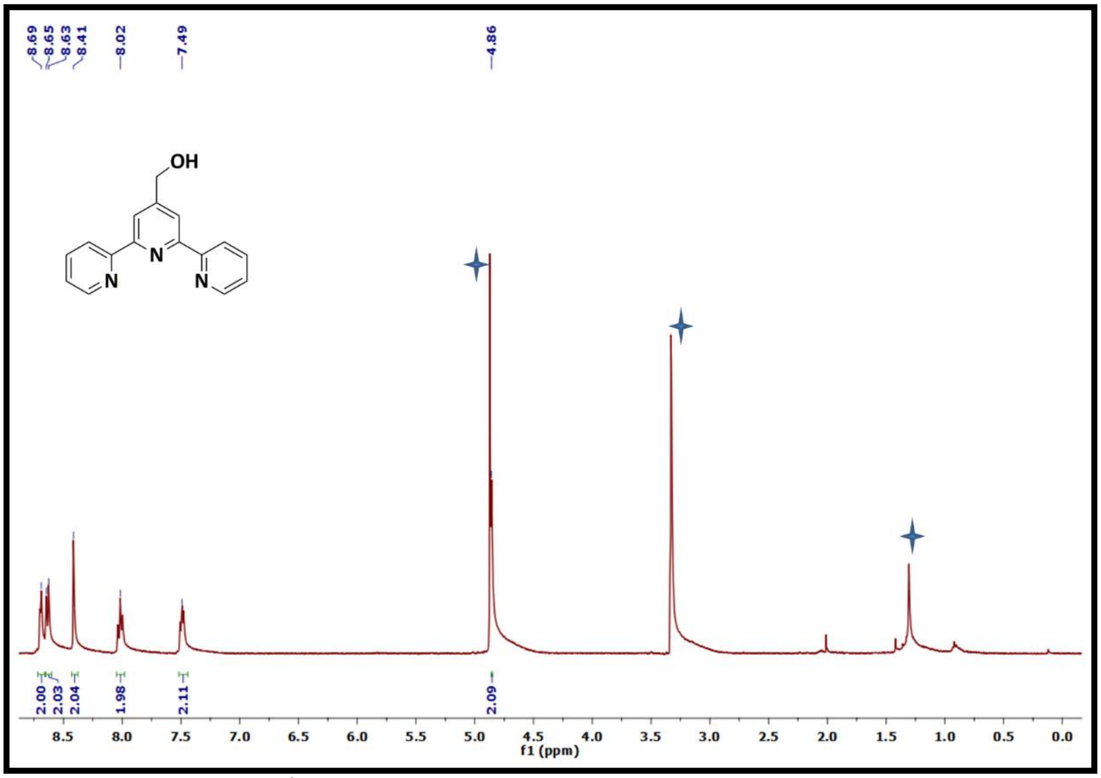

Supplementary Figure 8. ^{1}H-NMR Spectrum of Compound **4**.

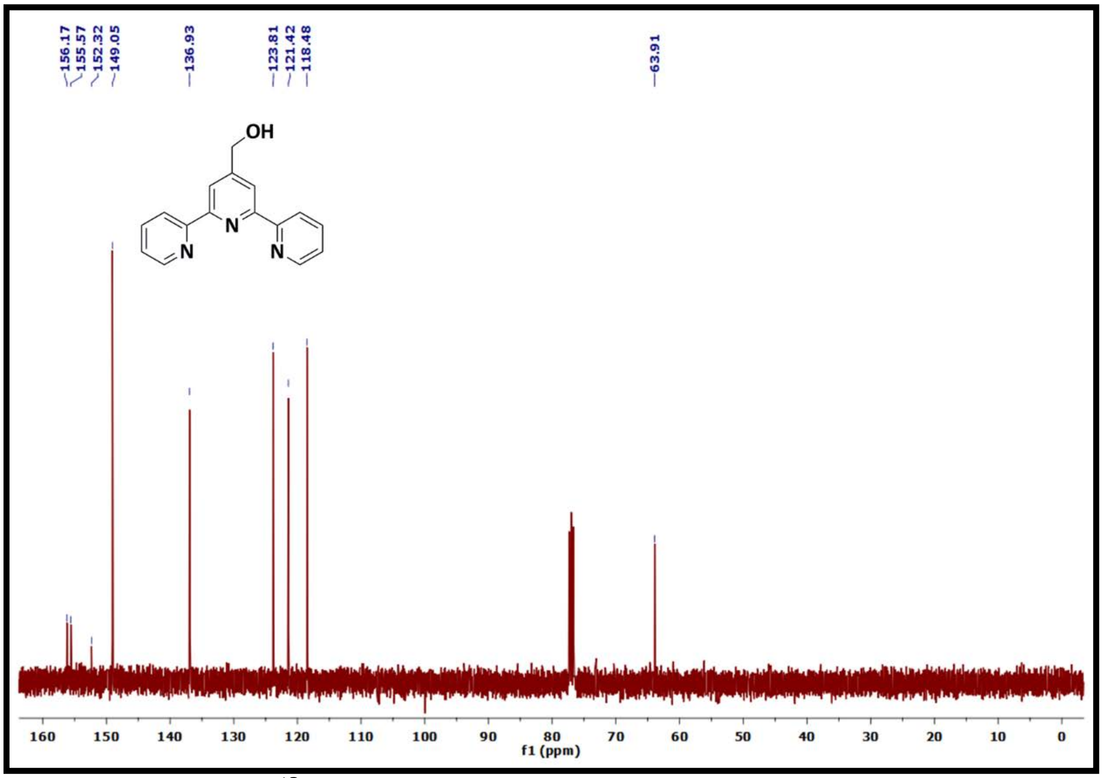

Supplementary Figure 9.^{13}C-NMR Spectrum of Compound **4**.

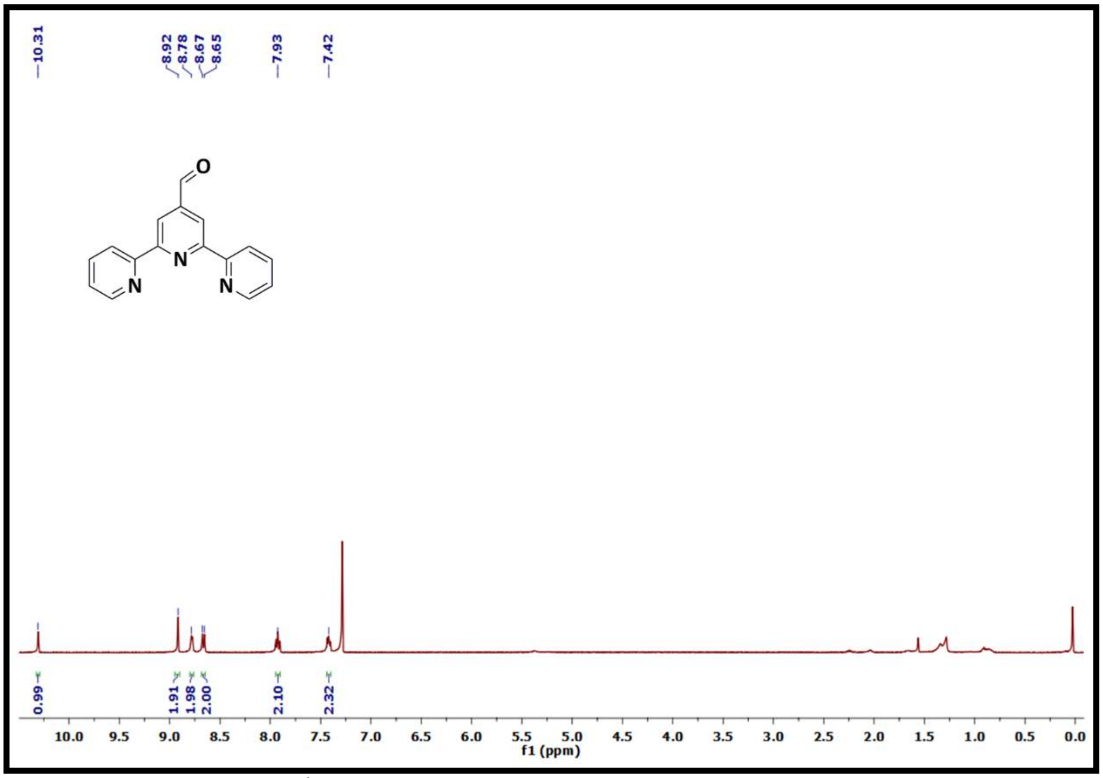

Supplementary Figure 10. ^{1}H-NMR Spectrum of Compound **5**.

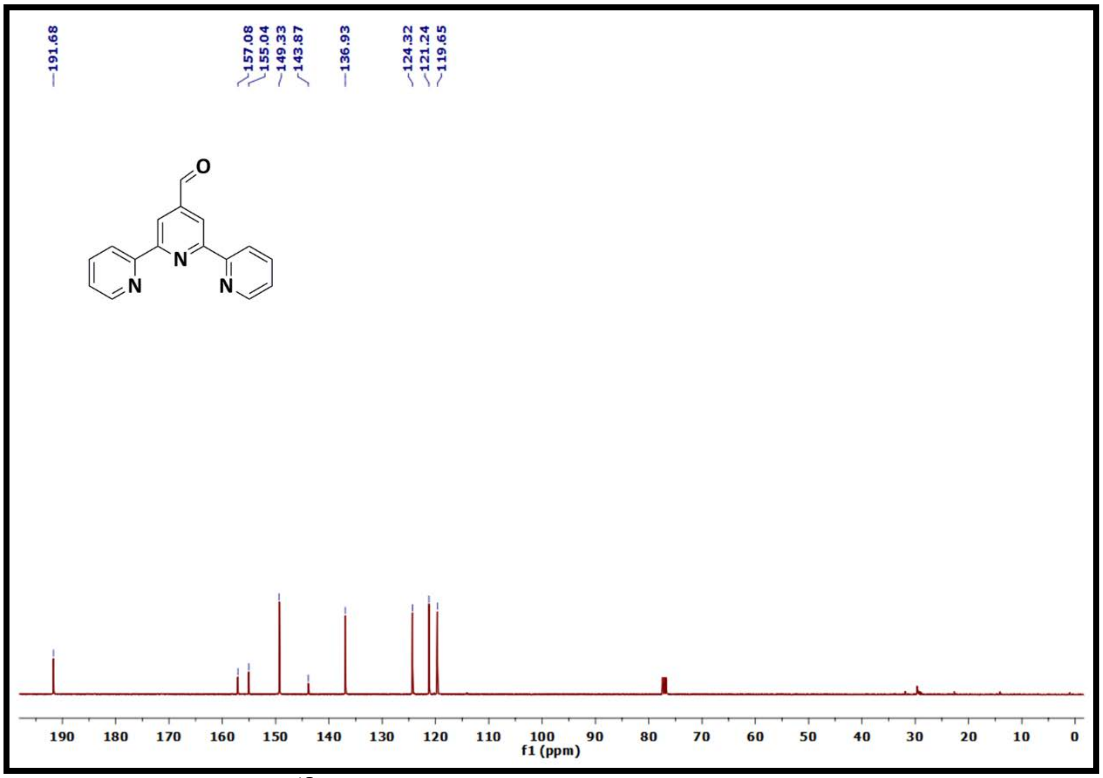

Supplementary Figure 11.^{13}C-NMR Spectrum of Compound **5**.

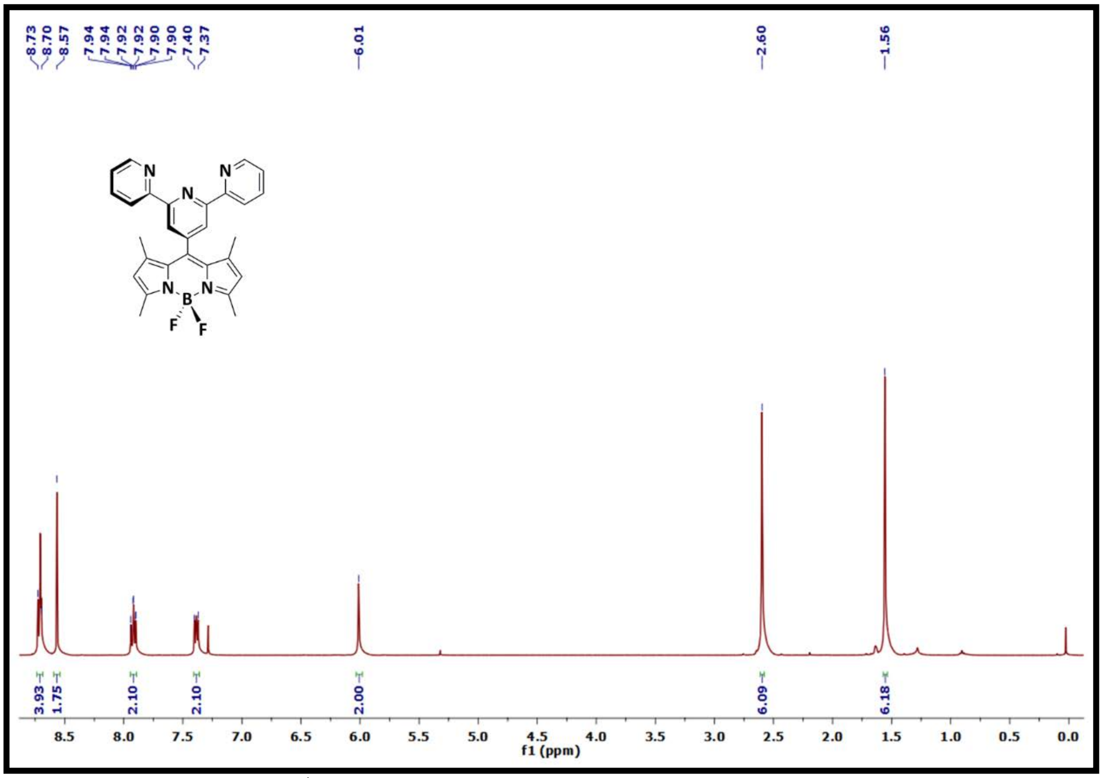

Supplementary Figure 12. ^{1}H-NMR Spectrum of Compound **6**.

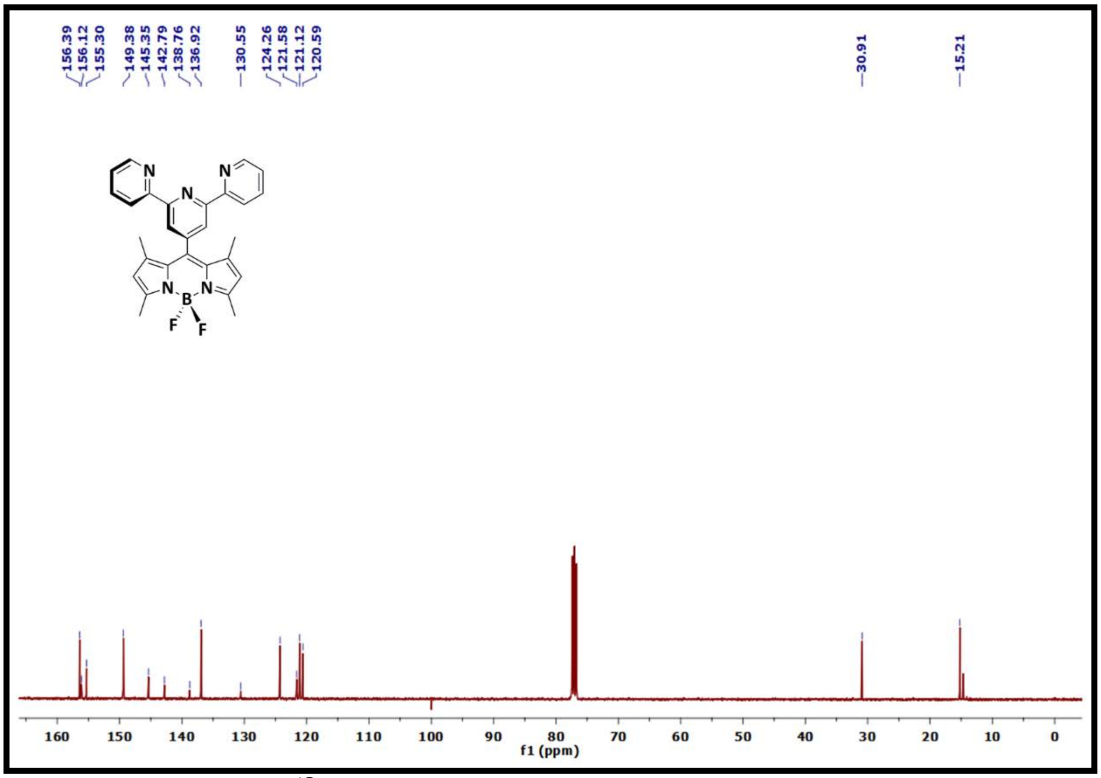

Supplementary Figure 13.^{13}C-NMR Spectrum of Compound **6**.

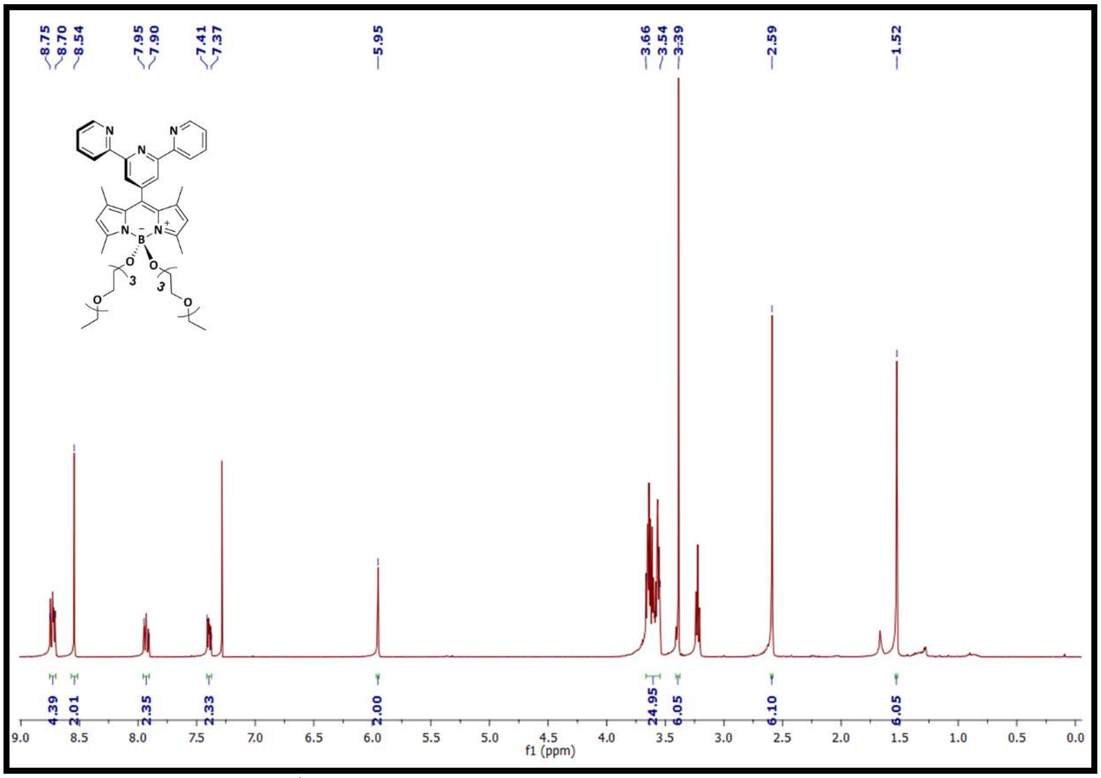

Supplementary Figure 14.^{1}H-NMR Spectrum of Compound **7**.

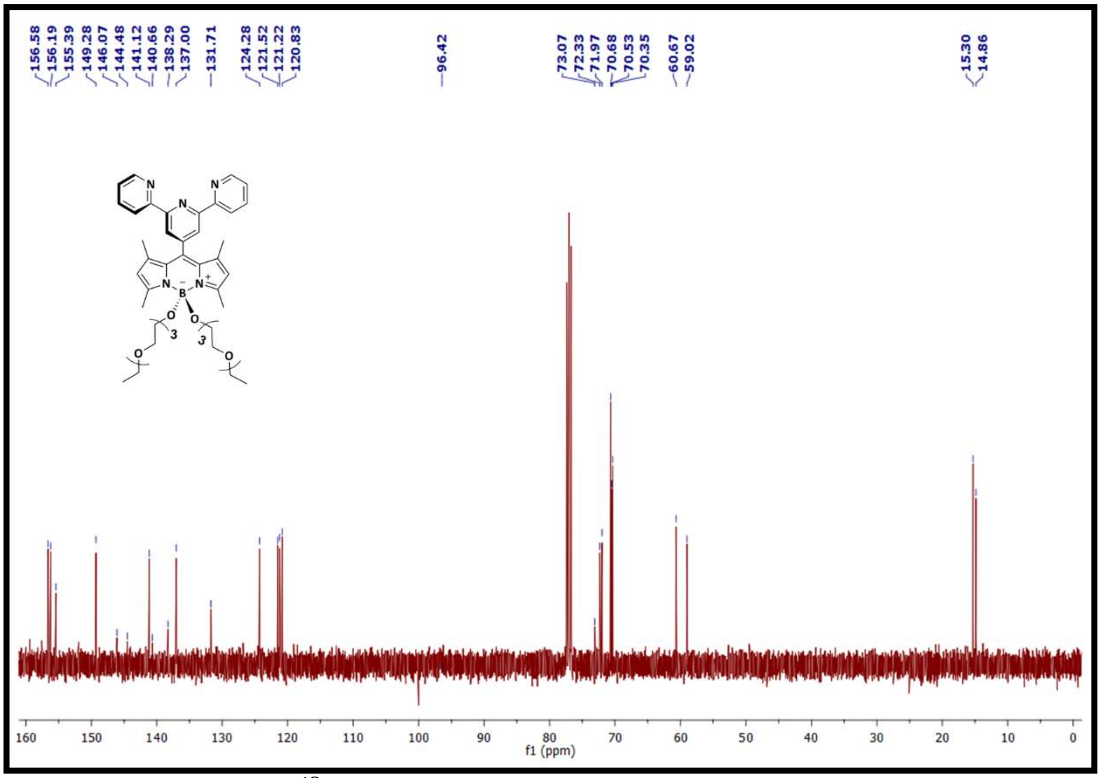

Supplementary Figure 15. ^{13}C-NMR Spectrum of Compound **7**.

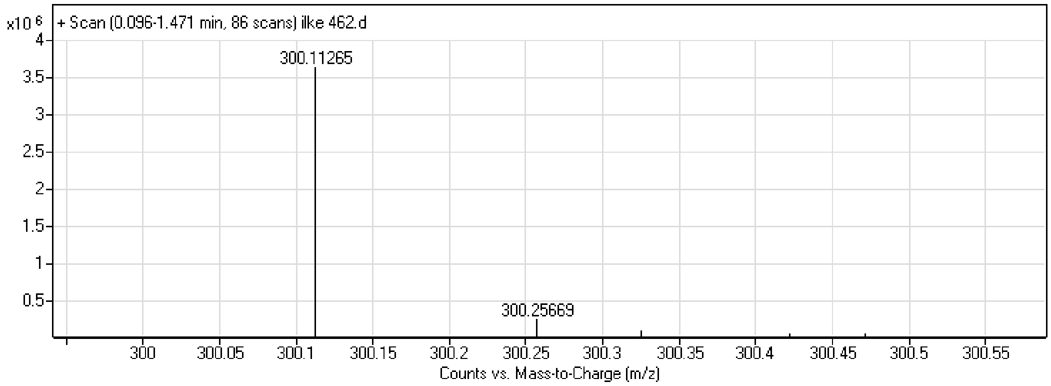

Supplementary Figure 16. HRMS Spectrum of Compound **1**.

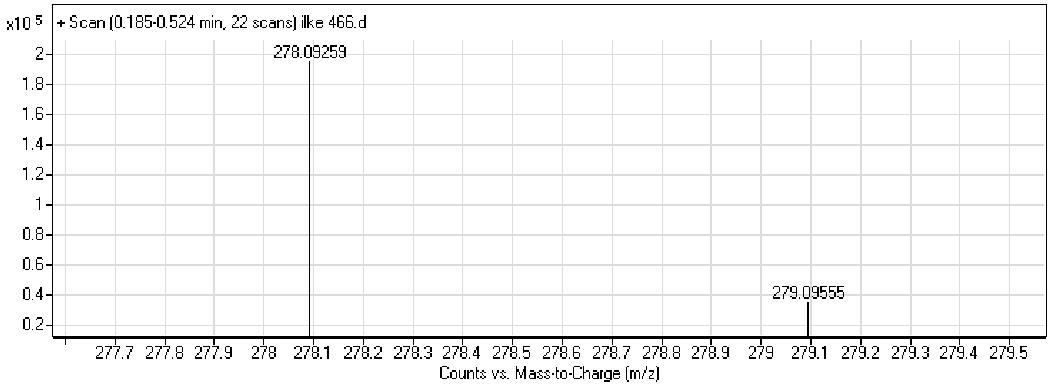

Supplementary Figure 17. HRMS Spectrum of Compound **2**.

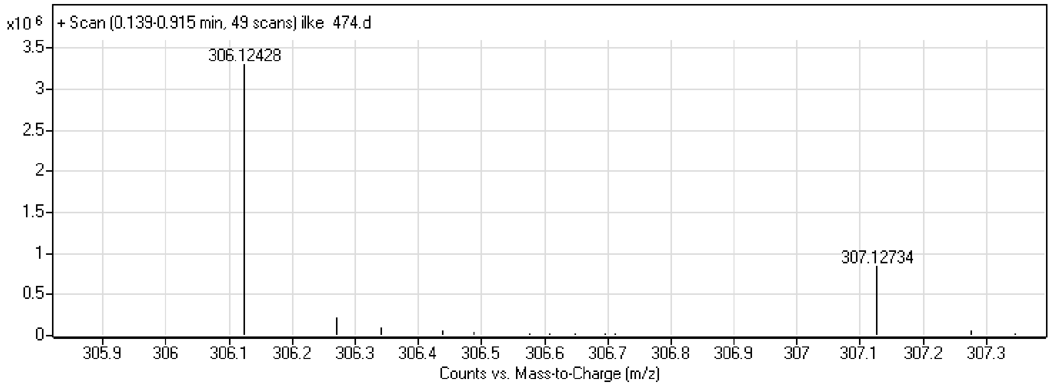

Supplementary Figure 18. HRMS Spectrum of Compound **3**.

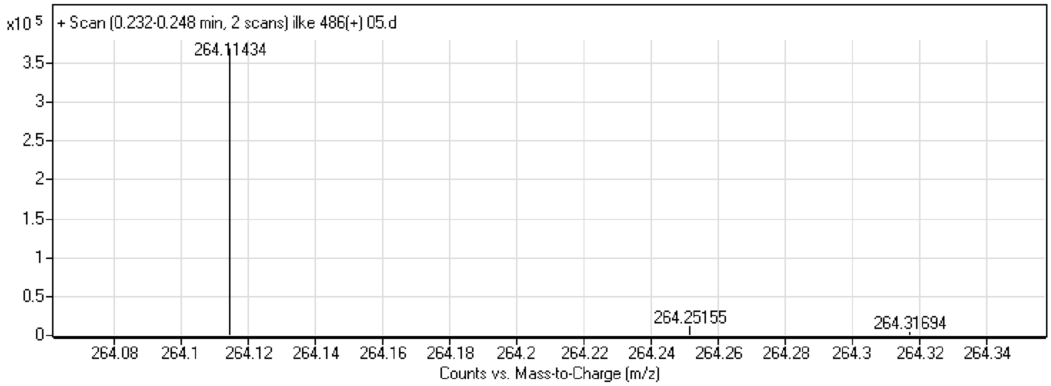

Supplementary Figure 19. HRMS Spectrum of Compound **4**.

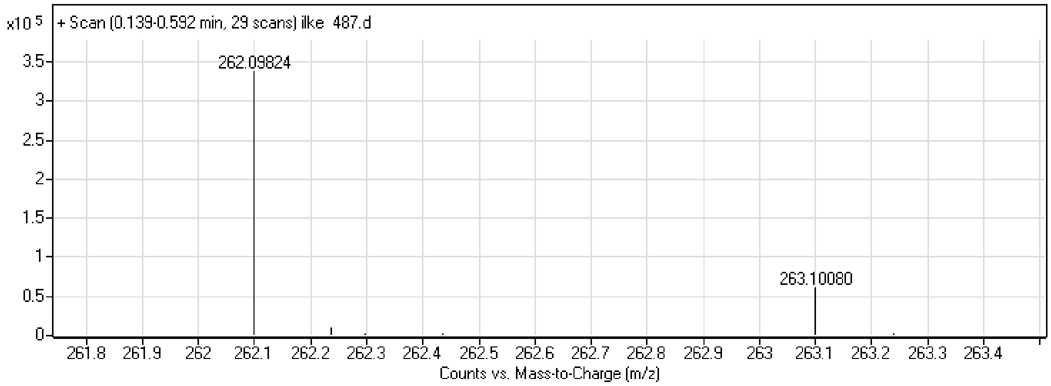

Supplementary Figure 20. HRMS Spectrum of Compound **5**.

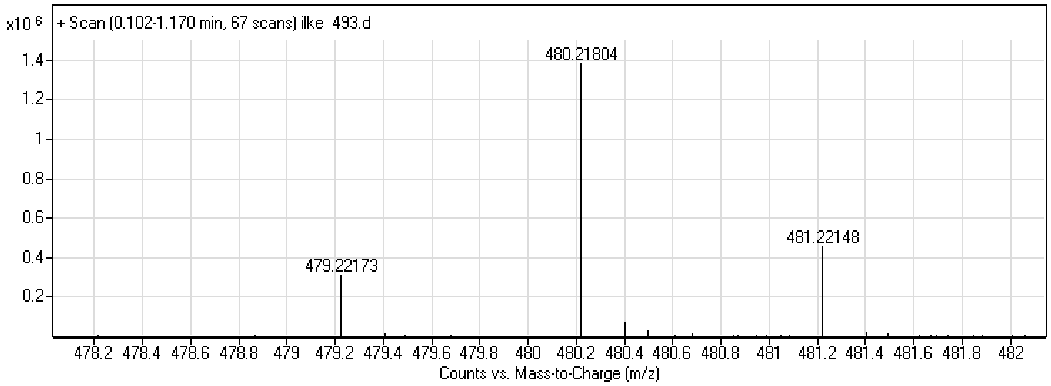

Supplementary Figure 21. HRMS Spectrum of compound **6**.

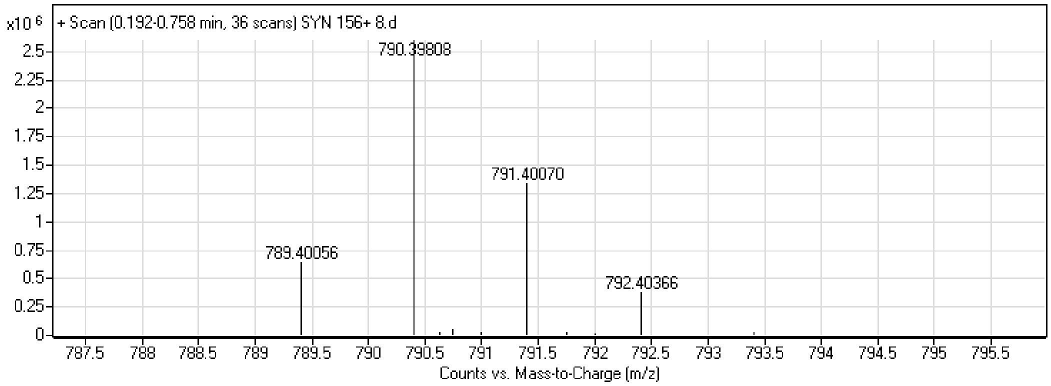

Supplementary Figure 22. HRMS Spectrum of compound **7**.

Spectroscopic and Photophysical Characterizations

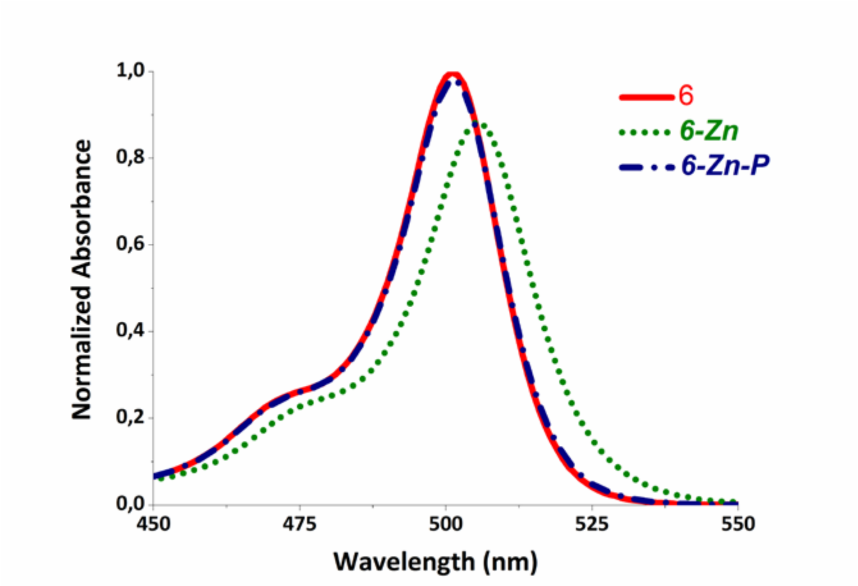

Supplementary Figure 23. Normalized Electronic Absorption Spectra of (***6***), (**6-*Zn***), (***6-Zn-P***) in CH_3CN wherein concentrations are 2µM for ***6***, 10 µM for ***Zn*** and 30 µM for ***P***.

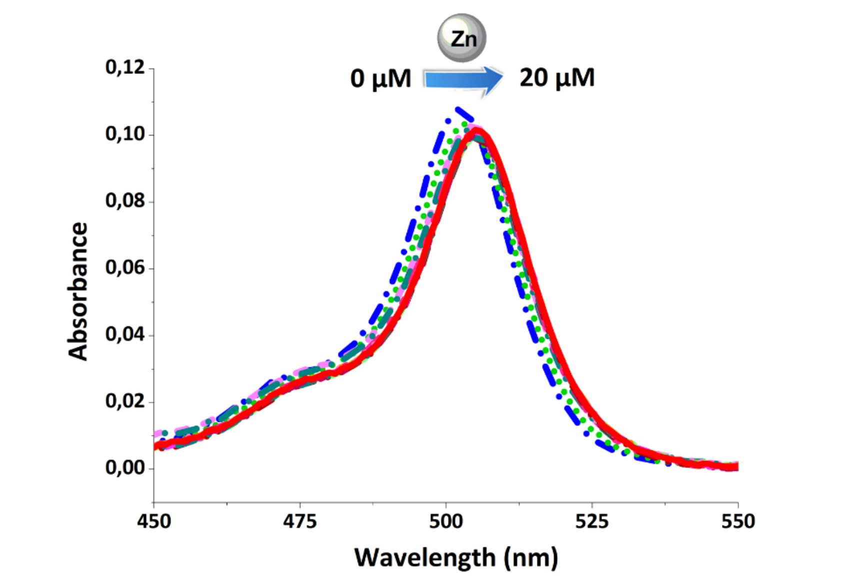

Supplementary Figure 24. Electronic Absorption Spectra of Bodipy ***6*** (2µM) upon increased Zn concentrations (0-10equiv.) in CH_3CN.

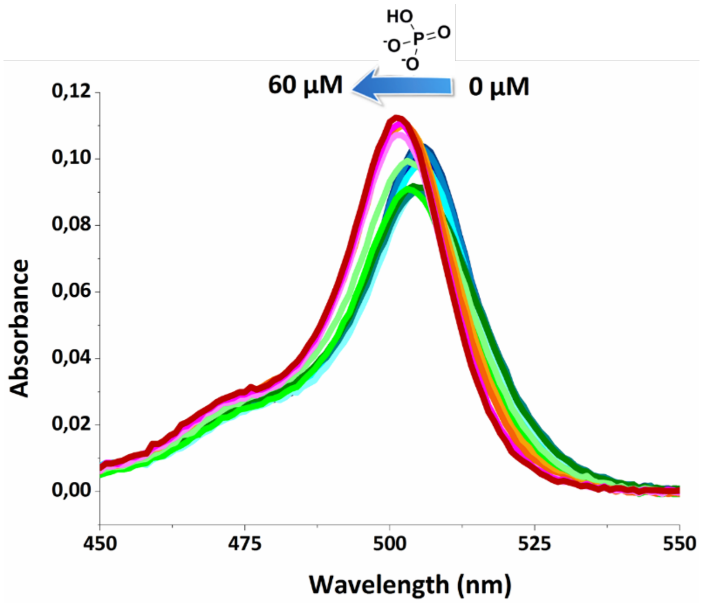

Supplementary Figure 25. Electronic Absorption Spectra of Bodipy **6** (2μM) –***Zn*** (5equiv.) conjugate upon increased concentrations of ***P***(0-30equiv.) in CH_3CN.

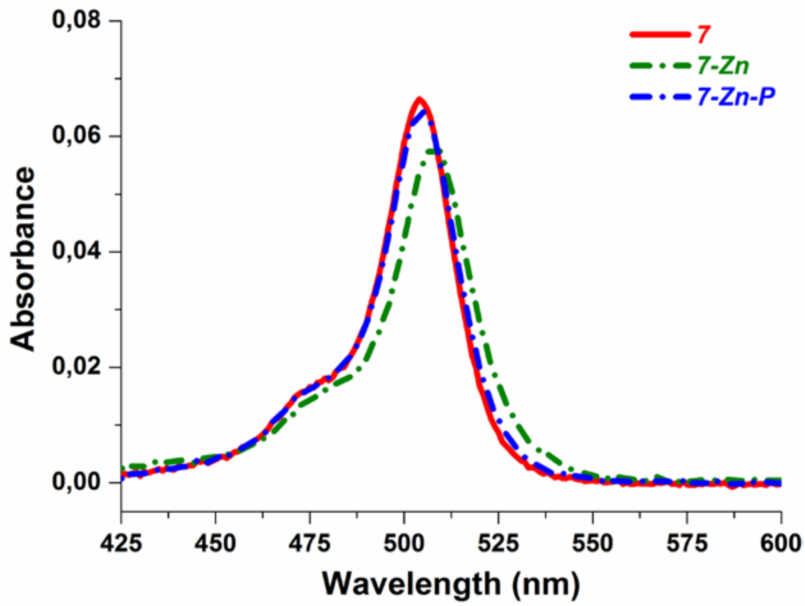

Supplementary Figure 26. Normalized Electronic Absorption Spectra of (**7**), (**7-Zn**), (**7-Zn-P**) in CH_3CN wherein concentrations are 1 μM for ***7***, 2 μM for ***Zn*** and 7 μM for ***P***.

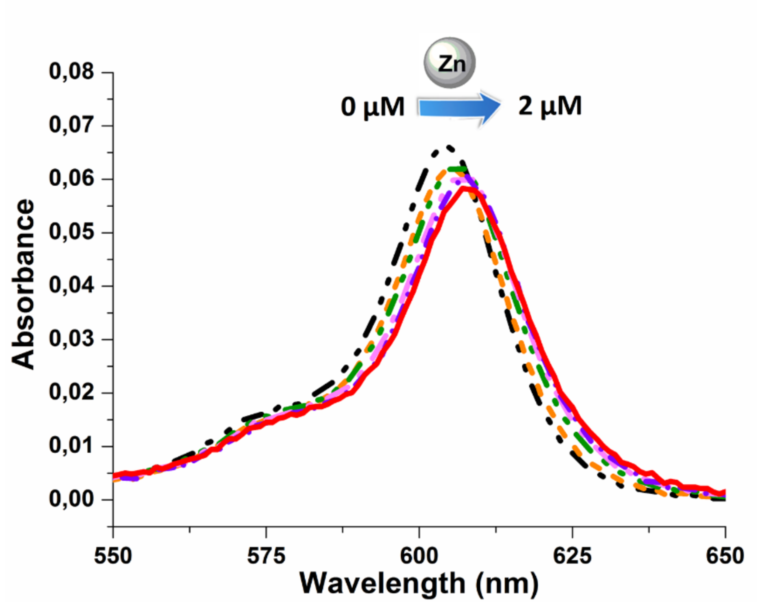

Supplementary Figure 27. Electronic Absorption Spectra of Bodipy ***7*** (1 µM) upon increased ***Zn*** concentrations (0-2 equiv.) in CH_3CN.

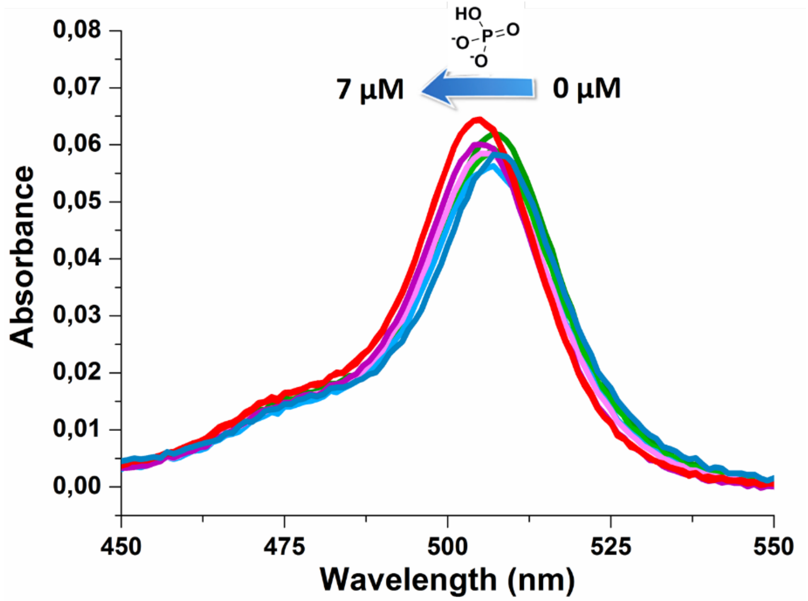

Supplementary Figure 28. Electronic Absorption Spectra of Bodipy **7** (1 µM) ***–Zn*** (2 equiv.) conjugate upon increased concentrations of ***P***(0 -7 equiv.) in CH_3CN.

Fluorescence Spectroscopic Measurements:

The fluorescence quantum yield (Φ_F) of the samples was calculated by using Supplementary Equation 1:

$$\Phi_F = \Phi_F^0 \frac{I}{I_0} \frac{A}{A_0} \frac{n^2}{n_0^2} \quad \textbf{(1)}$$

wherein ***I*** is the integrated fluorescence intensity, A is the absorbance at excitation wavelength, n is the refractive index of the solvent used, the subscript "0" stands for a reference compound. Rhodamine 6G was used as the reference compound (Φ_f=0.95 in water). Bodipy dyes (***6*** and ***7***) were dissolved in CH_3CN. Bodipy dyes and reference compound were prepared with the same absorbance (A_i) at the excitation wavelength (less than 0.05 in a 1.0 cm quartz cell). Reference compound and bodipy dyes (***6*** and ***7***) are excited at 488 nm. Calculated fluorescence quantum yields were given in the Supplementary Table 1.

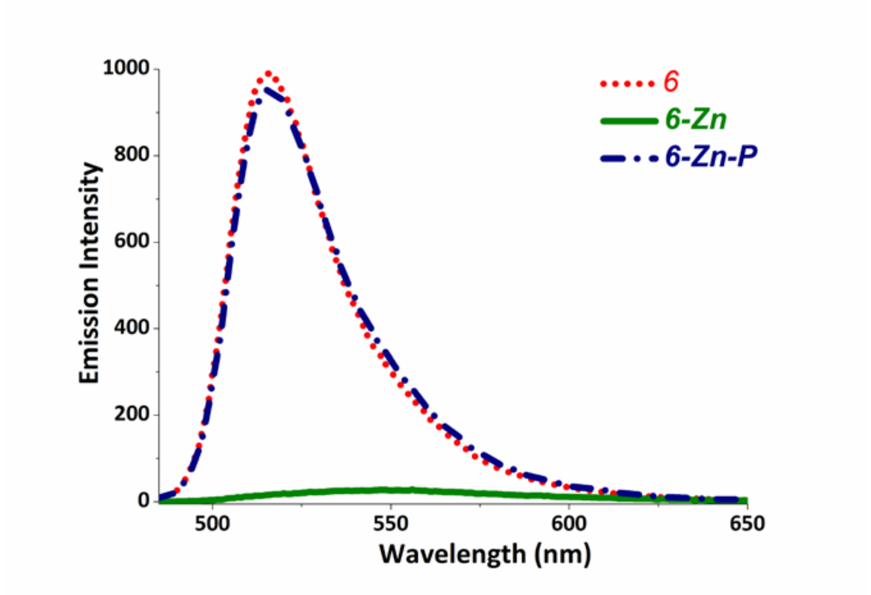

Supplementary Figure 29. Normalized Fluorescence Emission Spectra of (**6**), (**6-Zn**), (**6-Zn-P**) in CH_3CN wherein concentrations are 2 µM for ***6***, 10 µM for ***Zn*** and 30 µM for ***P***.

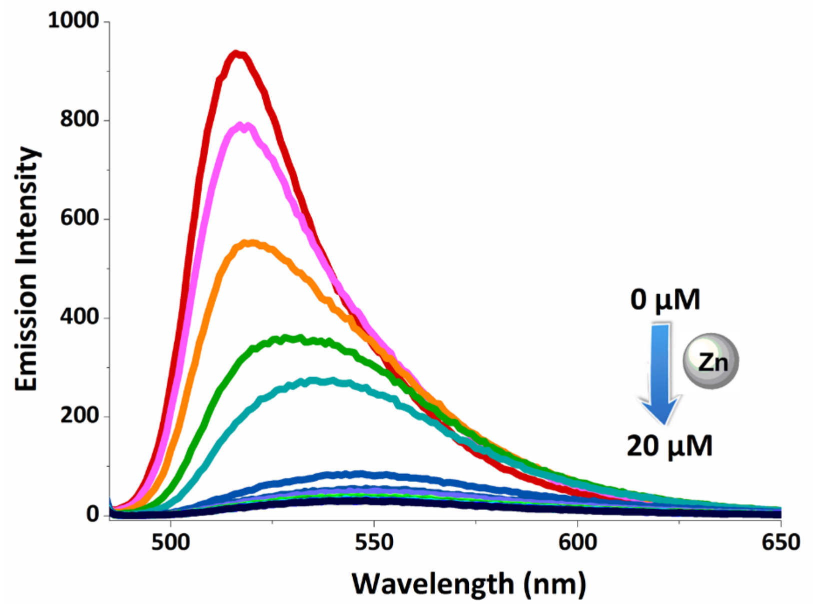

Supplementary Figure 30. Fluorescence Emission Spectra of Bodipy ***6***(2 µM) upon increased ***Zn*** concentrations (0-20 equiv.) in CH_3CN (λ_{ex}= 475 nm at 25 ^{0}C).

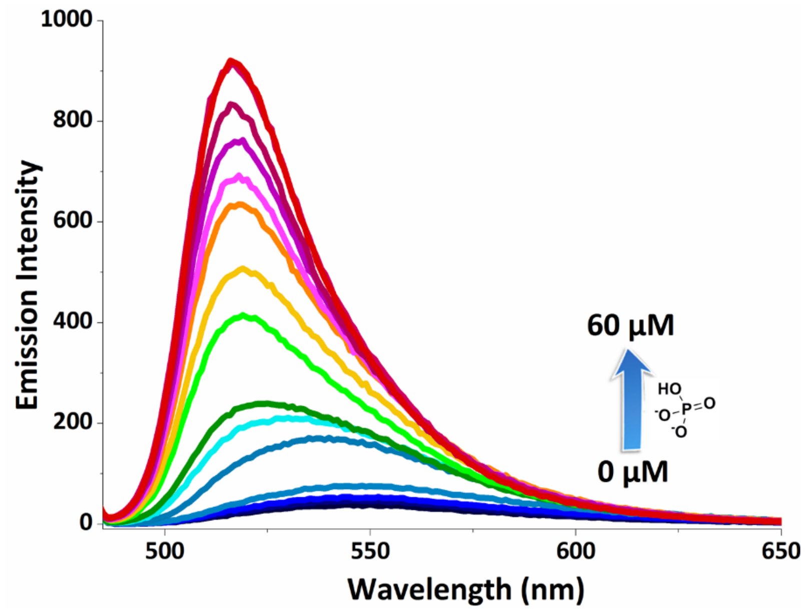

Supplementary Figure 31. Fluorescence Emission Spectra of Bodipy ***6***(2 µM)- ***Zn*** (5 equiv.) conjugate upon increasing concentrations of***P*** (0 -30 equiv.) in CH_3CN (λ_{ex}= 475 nm at 25 ^{0}C).

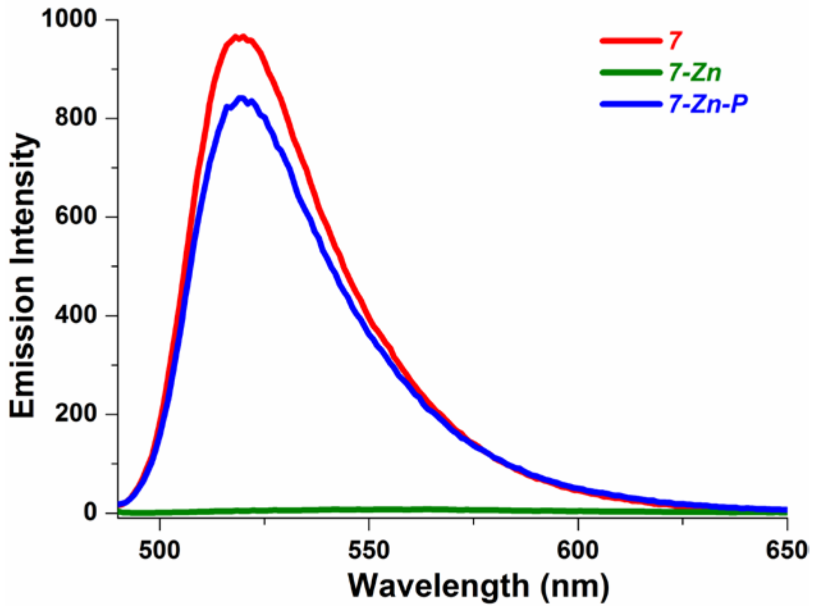

Supplementary Figure 32. Normalized Fluorescence Emission Spectra of (**7**), (**7-Zn**), (**7-Zn-P**) in CH_3CN wherein concentrations are 1 µM for ***7***, 2 µM for ***Zn*** and 7 µM for ***P***.

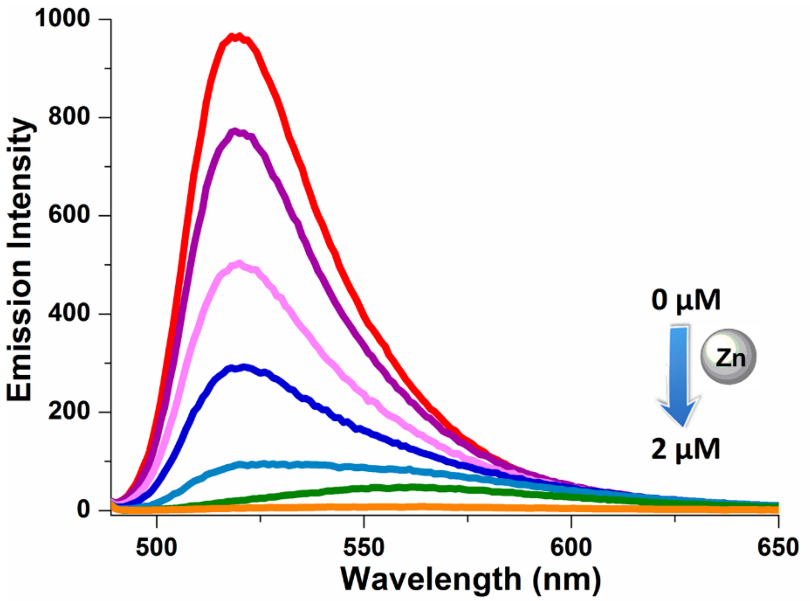

Supplementary Figure 33. Fluorescence Emission Spectra of Bodipy **7** (1 µM) upon increased ***Zn*** concentrations (0-2 equiv.) in CH_3CN (λ_{ex}= 480 nm at 25 ^{0}C).

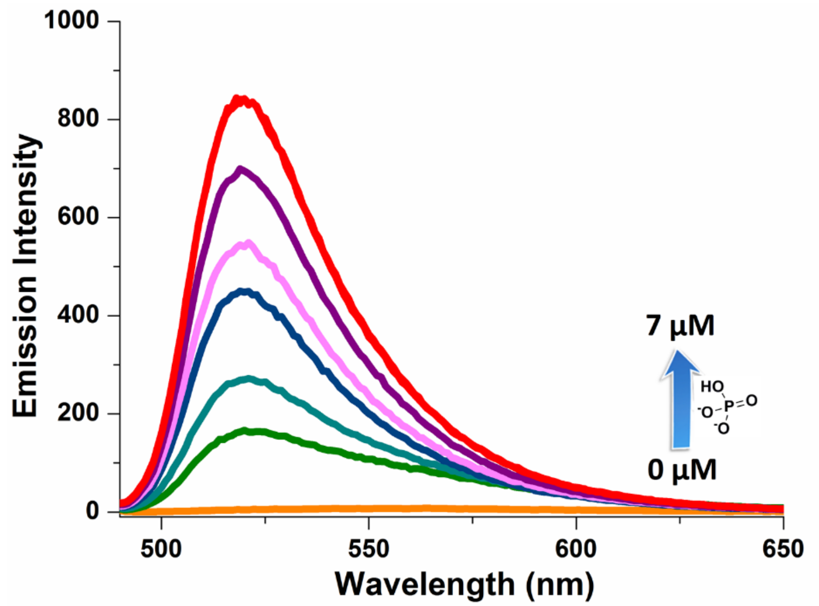

Supplementary Figure 34. Fluorescence Emission Spectra of Bodipy **7** (1 µM)- ***Zn*** (2 equiv.) conjugate upon increasing concentrations of***P*** (0 -7 equiv.) in CH_3CN (λ_{ex}= 480 nm at 25 ^{0}C) .

Supplementary Table 1. Photophysical data for (***6***), (***6-Zn***), (***6-Zn-P***), (***7***), (***7-Zn***), (***7-Zn-P***)

**Compound**[a]	**λ_{abs}(nm)**	**λ_{ems}(nm)**[b]	**ε** [c]	**Φ_F**[d]	**τ (ns)**	**Φ_Δ**[e]
**Bodipy 6**	501	517	67 000	0.34	1.62	-
6-Zn	505	547	59 200	0.048	**-**	0.11
**6-Zn-P**	501	517	65 800	0.37	1.56	-
**Bodipy 7**	503	520	66 400	0.49	-	-
7-Zn	507	558	58 200	0.082	3.41	0.10
**7-Zn-P**	505	518	64200	0.48	-	-

[a] Data acquired in CH_3CN.[b] λ_{exc}: 475 nm for Bodipy 6, λ_{exc}: 480 nm for Bodipy **7**. [c] Unit: cm^{-1} M^{-1}. [d] Relative quantum yields. Reference dye: Rhodamine 6G in water (Φ_f=0.95). [e] Relative singlet oxygen quantum yields. Reference dye: Eosin Y in ethanol.

Singlet Oxygen Trap Experiments:

In singlet oxygen measurements 1,3-Diphenylisobenzofuran (DPBF) was used as a singlet oxygen trap in CH_3CN and was purchased from a supplier. In a typical procedure for the detection of singlet oxygen generation by using trap molecules, a photosensitizer (~1 µM) and a trap molecule (O.D ~1.0) were mixed in O_2 bubbled CH_3CN. Initially several dark measurements were taken followed by irradiation of the mixture at absorption maximum of a sensitizer. Absorbance decrease of trap molecules was monitored suggesting singlet oxygen generation in the presence of light and sensitizers. Measurements were performed using 522 nm LED and samples were irradiated with the

light source from a 15 cm distance. All samples were homogenized for 1 min before measurements.

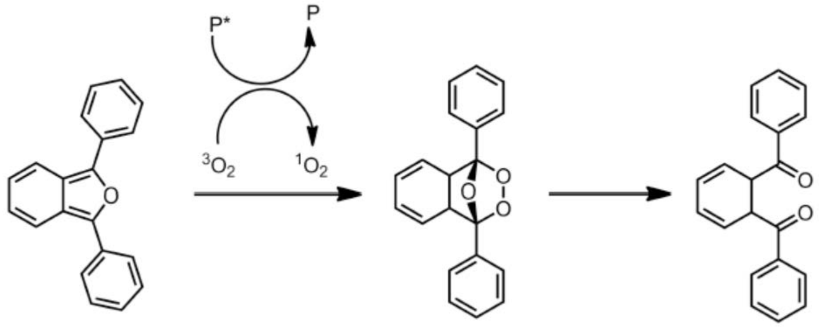

Supplementary Figure 35. Reaction of singlet oxygen with 1,3-Diphenylisobenzofuran.

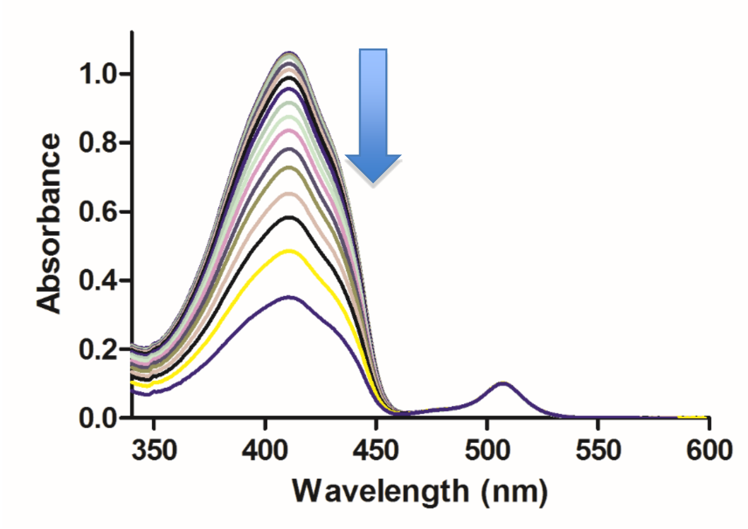

Supplementary Figure 36. Raw data for the decrease in absorbance of DPBF in ethanol in the presence of T2+Zn in medium. Details are given in Fig. 2 and Fig. 3. Singlet oxygen quantum yields were calculated as previously described using initial rate of decrease in the DPBF absorbance once any changes in absorbance of the DPBF in dark were subtracted[1]. The final curve (dark blue) is for 30 minutes.

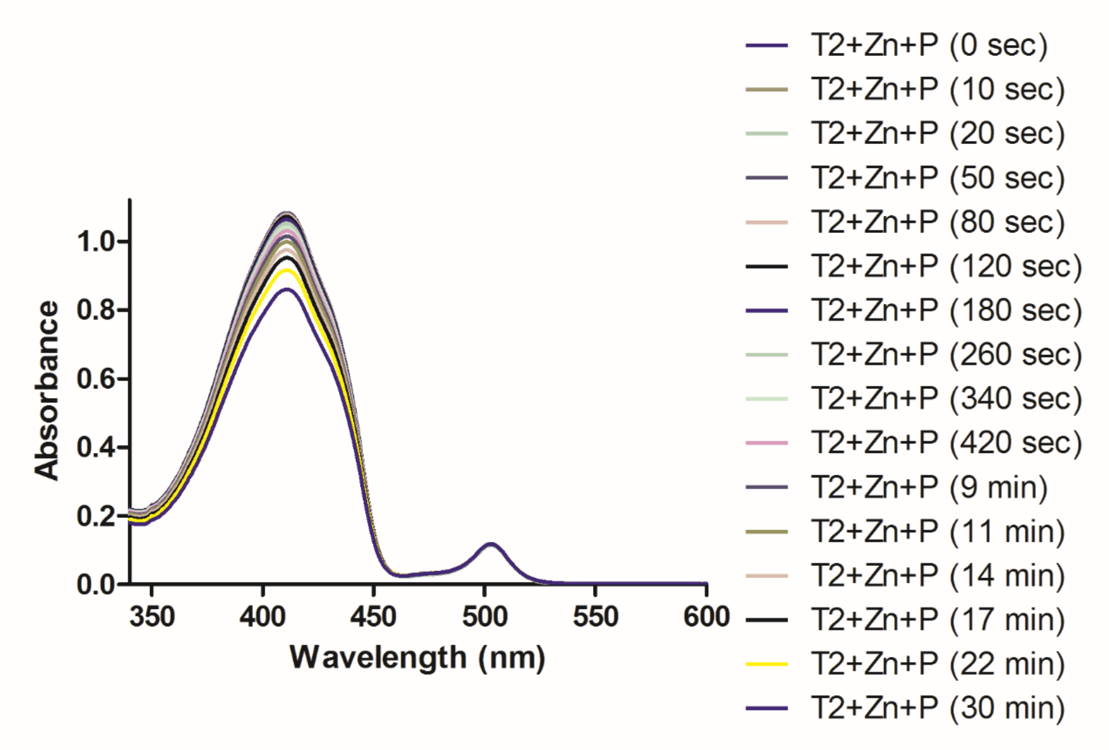

Supplementary Figure 37. Raw data for the decrease in absorbance of DPBF in ethanol in the presence of T2+Zn and phosphate in medium. Singlet oxygen quantum yields were calculated as previously described[1] using initial rate of decrease in the DPBF absorbance once any changes in absorbance of the DPBF without any agent added were subtracted[1]. In this particular case, the changes in absorbance is the same as the decrease in absorbance of DPBF alone, under irradiation.

Supplementary References

1. Cakmak Y, *et al.* Designing Excited States: Theory-Guided Access to Efficient Photosensitizers for Photodynamic Action. *Angew. Chem. Int. Ed.* **123**, 12143-12147 (2011).
